# Allosteric Transitions of Supramolecular Systems Explored by Network Models: Application to Chaperonin GroEL

**DOI:** 10.1371/journal.pcbi.1000360

**Published:** 2009-04-17

**Authors:** Zheng Yang, Peter Májek, Ivet Bahar

**Affiliations:** 1 Department of Computational Biology, School of Medicine, University of Pittsburgh, Pittsburgh, Pennsylvania, United States of America; 2 Department of Physics and Astronomy, School of Arts & Sciences, University of Pittsburgh, Pittsburgh, Pennsylvania, United States of America; 3 Department of Computer Science, Cornell University, Ithaca, New York, United States of America; Stanford University, United States of America

## Abstract

Identification of pathways involved in the structural transitions of biomolecular systems is often complicated by the transient nature of the conformations visited across energy barriers and the multiplicity of paths accessible in the multidimensional energy landscape. This task becomes even more challenging in exploring molecular systems on the order of megadaltons. Coarse-grained models that lend themselves to analytical solutions appear to be the only possible means of approaching such cases. Motivated by the utility of elastic network models for describing the collective dynamics of biomolecular systems and by the growing theoretical and experimental evidence in support of the intrinsic accessibility of functional substates, we introduce a new method, *adaptive anisotropic network model* (*a*ANM), for exploring functional transitions. Application to bacterial chaperonin GroEL and comparisons with experimental data, results from action minimization algorithm, and previous simulations support the utility of *a*ANM as a computationally efficient, yet physically plausible, tool for unraveling potential transition pathways sampled by large complexes/assemblies. An important outcome is the assessment of the critical inter-residue interactions formed/broken near the transition state(s), most of which involve conserved residues.

## Author Summary

Most proteins are biomolecular machines. They perform their function by undergoing changes between different structures. Understanding the mechanism of transition between these structures is of major importance to design methods for controlling such transitions, and thereby modulating protein function. Although there are many computational methods for exploring the transitions of small proteins, the task of exploring the transition pathways becomes prohibitively expensive in the case of supramolecular systems. The bacterial chaperonin GroEL is such a supramolecular machine. It plays an important role in assisting protein folding. During its function, GroEL undergoes structural transitions between multiple forms. Here, we are introducing a new methodology, based on elastic network models, for elucidating the transition mechanisms in such supramolecular systems. Application to GroEL provides us with biologically significant information on critical interactions and sequence of events occurring during the chaperonin machinery and key contacts that make and break at the transition. The method can be readily applied to other supramolecular machines.

## Introduction

Many proteins assume more than one functional conformation, stabilized by ligand binding or changes in environmental conditions. A typical example is the bacterial chaperonin GroEL [1], a widely studied ATP-regulated molecular machine and member of heat shock protein Hsp60 family. GroEL assists in unfolding and refolding misfolded or partially folded proteins [2]–[4]. It is composed of two back-to-back stacked rings, each containing seven subunits of 60 kDa. Each subunit is, in turn, composed of three domains, equatorial (E), intermediate (I) and apical (A). GroEL works together with the co-chaperonin, GroES.

**Figure 1:**
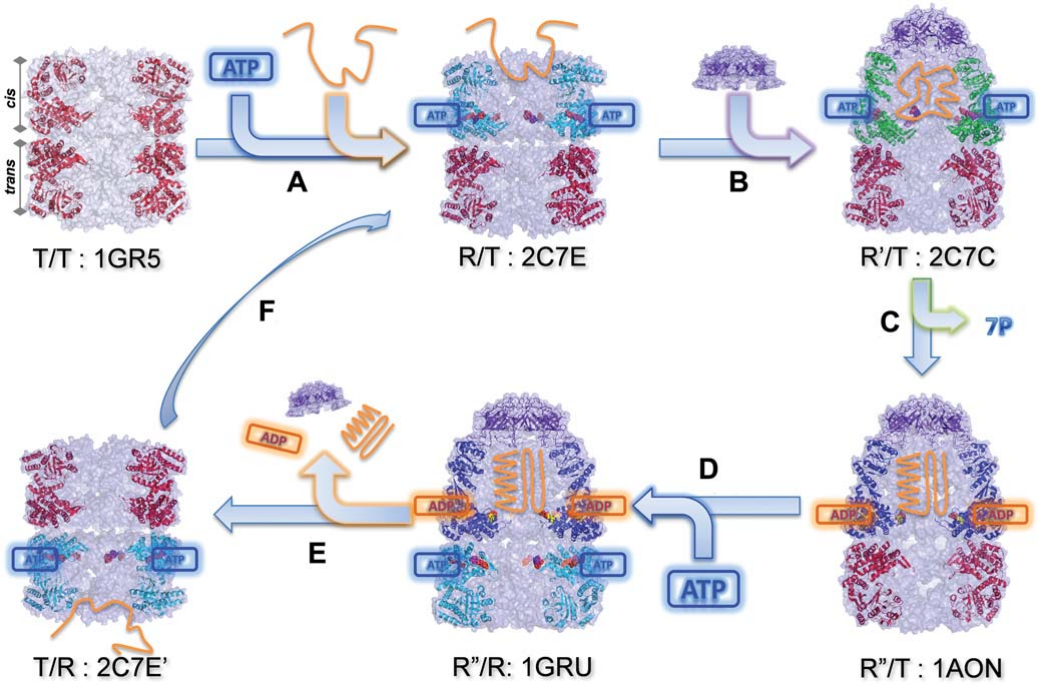
GroEL/GroES allosteric cycle. GroEL consists of two rings, *cis* and *trans*, which assume the states: T: ATP-free; R: ATP-bound prior to substrate (peptide) and co-chaperonin (GroES) binding; R′: ATP-, substrate- and GroES-bound; R″: ADP-, substrate- and GroES-bound. Subunits in the T state are shown in red, R in cyan; R′ in green, R″ in blue, and the cap in purple. ATP and ADP are shown by blue and orange boxes. Successive events/reactions along the cycle are (A) binding of seven ATPs to induce the binding of the unfolded substrate (orange), (B) co-chaperonin binding, (C) ATP hydrolysis, (D) ATP binding to *trans* ring subunits, (E) release of ADPs, substrate (folded or partially folded) and GroES from the *cis* ring, (F) initiation of a new cycle where the roles of the *cis* and *trans* rings are inverted. Top-middle and bottom-left structures are related by rigid body rotation. Diagrams were generated using the data from the PDB in PyMOL (http://www.pymol.org), except for the schematic views of the substrate and ligands included to provide a clearer description. The PDB ids for the structures T/T, R/T, R″/R, R′/T and R″/T are 1GR5, 2C7E and 1GRU [68], 2C7C [15] and 1AON [20], respectively.

The activity of GroEL-GroES complex entails a series of allosteric transitions in structure, triggered by ATP binding and hydrolysis, described in Figure 1: In the absence of nucleotide binding, both rings assume the closed (T) state, designated as T/T state for the two rings. Cooperative binding of seven ATP molecules to the subunits in one of the rings, hereafter referred to as the *cis* ring, drives the conformational change of these subunits to the ‘open’ (R) state, thus leading to the R/T form of the *cis/trans* rings. The R/T form exposes a number of hydrophobic residues at the apical domains of the *cis* ring subunits. These groups attract the unfolded or partially folded peptide (substrate) to be encapsulated in the cylindrical chamber following the attachment of GroES (R′/T form of *cis/trans* rings). ATP hydrolysis provides the energy needed to process (unfold/refold) the substrate and leads to the state R″/T. This process is terminated upon binding of seven ATP molecules to the adjoining (*trans*) ring, hence the term ‘negative cooperative effect’ induced by ATP binding. The structure with ADP and ATP molecules bound to the respective *cis* and *trans* rings (R″/R form) favors the opening of the GroES cap and release of the peptide and ADP molecules to start a new cycle, this time, with the roles of the former *cis* and *trans* rings being inverted.

Of interest is to understand the molecular basis of the negative cooperative effect triggered upon binding of seven ATP molecules to the *trans* ring (step E in Figure 1). ATP binding induces in this case a structural change on the cap-binding region at a distance of 65 Å. Understanding the mechanism of this allosteric signaling is of fundamental importance because of the critical role chaperonins play in preventing aggregation and regulating folding *vs.* degradation events.

Several studies have been published on the allosteric pathways and dynamics of GroEL [3]–[19] since the original determination of the ADP-bound complex (R″/T) [20]. These studies provide valuable insights into the successive states involved in the chaperonin cycle. The elucidation of allosteric transition mechanisms has been a challenge, however, both experimentally and computationally, due to the transient nature of the high energy conformers between the states, the multiplicity of pathways, and the large size of this biomolecular system.

### Computational methods for exploring transition pathways

Several computational methods have been developed in the last two decades for exploring the structural transition pathways of biomolecules (for reviews see [21],[22]). These include methods based on minimizing path-dependent functionals [23],[24], stochastic path integration [25], MaxFlux method for identifying the path of maximum flux [26],[27], nudged elastic band method [28], and the determination of temperature-independent reaction coordinates by action minimization [29]–[31]. Other groups resorted to targeted molecular dynamics (TMD) simulations in the presence of holonomic constraints [11], [32]–[36], Monte Carlo- and MD-based methods for sampling ensembles of stochastic transition paths (e.g., [37],[38] and perturbation-targeted MD combined with umbrella sampling [39],[40]). Yet, the identification of the transition state(s) and accompanying conformational rearrangements are by and large inaccessible for systems of the order of megadaltons like GroEL. Coarse-grained models and methods appear as the only tractable tools in such cases. Perhaps the most comprehensive computational study of GroEL allosteric dynamics is that of Hyeon, Lorimer and Thirumalai, where the T→R→R″ transition has been examined by Brownian dynamics (BD) simulations using a state-dependent (Go-like) self-organized polymer model [18]. These simulations, performed for subunit dimers and heptamers, provided valuable insights on the heterogeneity of the transition pathways and the kinetics of salt bridges' formation/rupture at the successive transitions T→R and R→R″.

### Elastic Network Models (ENMs)

Recent years have seen a revival in normal mode analyses (NMAs) of proteins, after realizing that coarse-grained models such as the ENM provide information on functional and robust modes [41]–[45]. ENMs have been recently used for exploring the transition pathways between known end conformations: Kim *et al.* proposed to interpolate between the end points by controlling the *distances* between the nodes, rather than the position vectors so as to avoid unrealistic deformation [46]. Onuchic and coworkers extended the concept of minimal frustration to transitions between basins modeled as ENMs, and examined the coupling between the strains in dihedral angles and local unfolding events (also termed *cracking*) [47],[48]. Maragakis and Karplus explored the transition of adenylate kinase between its T and R forms using a combination of steepest descent and conjugate peak refinement algorithms [49]. More recently, Chu and Voth presented a double-well ENM model for exploring the conformational transitions of G-actin and adenylate kinase [50].

The success of these studies suggests that ENM-based methods may serve as a first approximation for exploring the transitions between not-too-distant pairs of functional states. Here we present a new approach based on the anisotropic network model (ANM) [51],[52] to this aim, and apply it to the allosteric transitions of the GroEL-GroES complex (∼8,000 residues, or ∼1 MDa). The approach, referred to as ***a*** **daptive ANM** (***a*** **ANM**), utilizes the ANM modes to guide the complex along the directions intrinsically favored by its instantaneous inter-residue contact topology.

The ANM is based on the following assumptions: (i) the experimentally resolved structures are assumed to represent an energy minimum, (ii) the structures are modeled as a network, the nodes of which represent the individual amino acids, (iii) the positions of the nodes are identified by those of the α-carbons, (iv) all pairs of nodes separated by a distance shorter than a cutoff distance of r_c_ are connected by an elastic spring, (v) a uniform force constant is assumed for all pairs regardless of amino acid type. Thus, the structure is subject to a sum of harmonic potentials corresponding to all connected (bonded or non-bonded) amino acids. The structural coordinates are used to derive the *3N-6* orthonormal modes of motion, called ANM modes, uniquely defined for each equilibrium structure of *N* modes [51],[52]. The ANM is now widely used in exploring protein dynamics and assessing the global (lowest frequency modes) in particular, in view of the observed robustness and functional relevance of these modes [41]–[45], [53]–[55]. In the present study, the potential of the system is approximated by the sum of two harmonic ANM potentials adopted for the end states, along with a softening term that ensures a smooth transition between them. As will be explained below, the softening term (and the corresponding parameter *β*; see Methods) is not used in the *a*ANM generation of the pathways, but in the comparison of the results against those predicted by other methods.

Multiple paths (or subsets of ANM modes) exist for the passage between the endpoints. The recruitment of the particular subsets of modes results from a tradeoff between minimizing the path length and selecting the direction of the lowest increase in internal energy. The ‘shortest’ path is by definition the interpolation between the two endpoints. However, this path usually incurs unphysical strains in internal coordinates, and is, thereby, unfavorable from energetic point of view. In the other extreme case of movements along the lowest-lying (softest) modes, on the other hand, these particular mode directions may not necessarily lead to the target. The selected pathway will thus depend on the relative importance of environmental (*path length*) or internal (*conformational energy barrier*) constraints (see below).

The results are organized in two sections: First (*Section I*), we present the results for the R″←→T transition of a single GroEL subunit (*N* = 514 residues). These calculations serve two purposes: benchmarking our results against those obtained by action minimization method [29], and estimating the effect of different choices of parameters. These data are then utilized in the second part (*Section II*) where we examine the intact chaperonin allosteric cycle and compare with experimental data and other simulations. Notably, a small subset of low frequency modes are found to drive the transition at early stages, succeeded by the recruitment of increasingly larger subsets of modes (yet not exceeding 4% of the entire spectrum) to overcome the energy barrier. Native contacts are closely maintained throughout significant portions of transition pathways, except for the close neighborhood of the energy peak, also called putative transition state (PTS), where a major redistribution in intra- and inter-subunit contacts takes place. The results provide new insights and testable hypotheses on the mechanistic involvement of conserved residue pairs in critical interactions.

### Theory: Adaptive ANM (aANM)

#### Definitions

The method is based on the simultaneous generation of pairs of intermediate conformations, 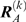 and 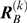, starting from the known endpoints 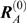 and 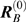. Each conformation along the so-called reaction coordinate is represented by a *3N*-dimensional vector, corresponding to the coordinates of α-carbons. The *distance vector* ***d*** ^(*k*)^ between the pair of conformations generated at iteration *k* is defined as
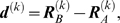
(1)and the *deformation vectors* used to generate the *k^th^* pair of conformations are
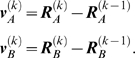
(2)


The original distance vector between the two optimally superimposed end structures is 
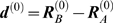
. This distance vector also defines the original root-mean-square deviation (RMSD), |***d*** ^(0)^|/*N*, between the endpoints. The RMSD at iteration *k* is similarly defined as
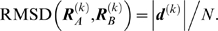
(3)


#### Methodology

**Figure 2:**
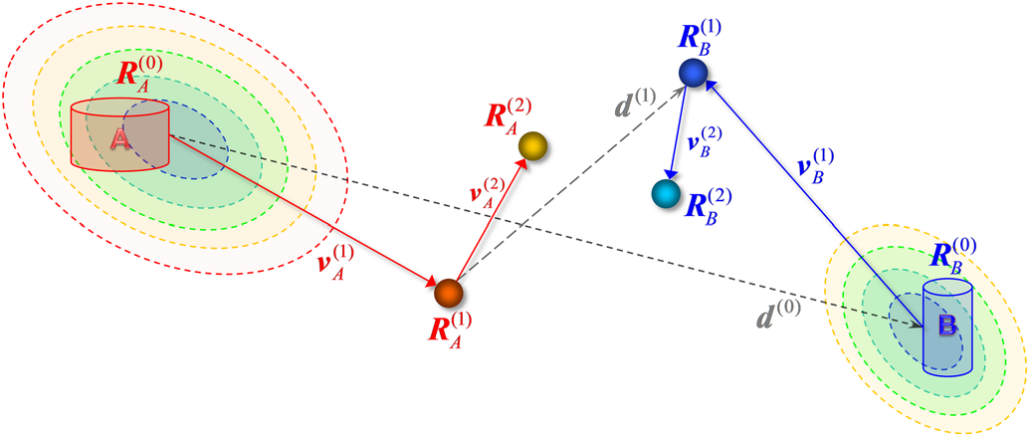
Schematic description of *a*ANM method. Two sets of intermediate conformations are generated, 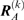 and 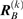 (1≤*k*≤*k_tot_*), starting from the known substates 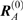 and 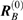, illustrated here for *k* = 1 and 2. The distance vector between the instantaneous endpoints at the *k^th^* step is denoted as 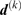, and the deformation at each step is 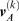 or 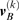. Dashed ellipses indicate isoenergetic contours.

The ***a*** **ANM** method consists of the following steps (see Figure 2):

(i) Two sets of intermediates are generated, starting from both ends. The recurrence equation for evaluating the *k^th^* intermediate starting from state *A* is
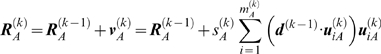
(4)and a similar expression holds for state *B*. Following Eq. (4), we evaluate 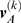 on the basis of 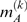 dominant (low frequency) eigenvectors 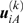 (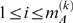) predicted by the ANM for the conformation 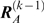. The displacement along the *i^th^* eigenmode is proportional to the projection 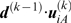 of the instantaneous distance vector 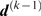 on 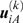, and scaled by the *step size* 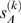. The step sizes *s_A_* ^(*k*)^ and *s_B_* ^(*k*)^ are simultaneously selected at each iteration *k*, as a fraction *f* of those, *s_A,m_* ^(*k*)^ and *s_Bm_* ^(*k*)^, that minimize ***d*** ^(*k*)^. The limit *f*→0 refers to infinitesimally small displacements that are strictly accurate but prohibitively expensive, while the other extreme case *f*→1 is the most efficient move, but may give rise to unphysical deformations in structural coordinates. *f* = 0.2 is selected here as a scaling factor that optimally balances between efficiency and accuracy (see *Methods*).

(ii) The number 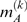 of modes of motion recruited at iteration *k* is based on a threshold squared cosine, *F_min_*, that defines the maximal angular departure between the instantaneous displacement direction and that targeted. To this end, we evaluate the *cumulative squared cosine*

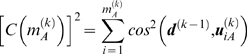
(5)and we select the minimal number of modes, starting from the low frequency end of the spectrum, that satisfy the inequality [*C*(*m_A_* ^(*k*)^)]^2^ *≥F_min_*. It can be shown (see derivation in Text S1) that *C*(*m_A_* ^(*k*)^) is identical to the correlation cosine between the instantaneous deformation and distance vectors, i.e.,
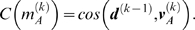
(6)


Therefore, the threshold *F_min_* ensures the selection of the *smallest* subset of modes to drive the deformation 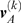 of the molecule toward a direction that does not deviate by more than a specified correlation cosine (*F_min_* ^½^ ) from the target direction ***d*** ^(*k−1*)^. Note that the use of the complete set of modes leads, by definition, to *C*(*3N-6*) = 1. By selecting a subset, we let the molecule undergo a structural change that is not necessarily toward the endpoint, but along the coordinates energetically favored by its architecture.

**Figure 3:**
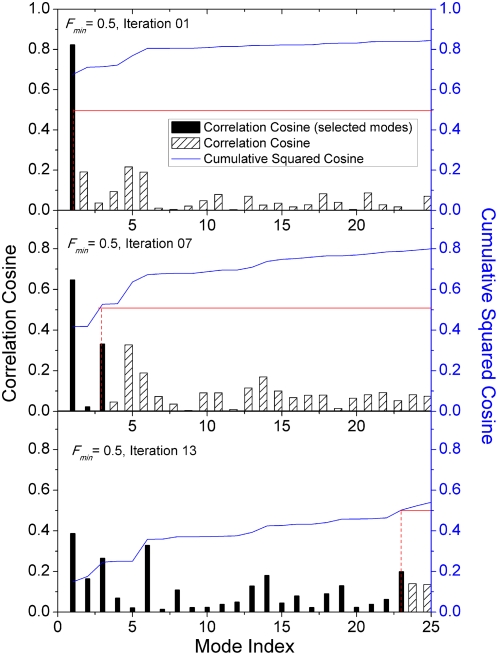
Correlation cosine between instantaneous distance vector and eigenmodes. Results are illustrated for *a*ANM steps *k* = 1, 7 and 13 along the transition R″→T of a single subunit (subunit A in the respective PDB structures 1GRU and 1GR5). The left ordinate displays the correlation cosine between the distance vector *d* ^(*k−1*)^ and the eigenvectors 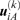 for 1≤*i*≤30 (black bars), and the right ordinate shows the corresponding cumulative squared cosine (Eq. (5)) (blue curve). The threshold *F_min_* = 0.5 for the cumulative square cosine implies the selection of *m_A_* ^(1)^ = 1, *m_A_* ^(7)^ = 3, and *m_A_* ^(13)^ = 23 in evaluating *v* *_A_* ^(*k*)^ as indicated by the red lines and filled bars. See Table 1 for the complete list of 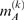 and 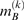 values and associated RMSDs between intermediate conformations.

Figure 3 illustrates the procedure for selecting *m_A_* ^(*k*)^ for the transition R″→T (step E in Figure 1). Results are shown for the *a*ANM iterations *k* = 1 (*top*), 7 (*middle*) and 13 (*bottom*). The bars displays the correlation cosine 
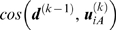
 as a function of mode number 1≤*i*≤25 (*left* ordinate), and the blue curve is the cumulative squared cosine [*C*(*m_A_* ^(*k*)^)]^2^ (*right*). For *k* = 1, the lowest frequency mode (*i* = 1) alone yields a correlation cosine of 0.82: it suffices, therefore, to take *m_A_* ^(1)^ = 1 mode at this step to meet the criterion [*C*(*m_A_* ^(*k*)^)]^2^≥*F_min_* , if the threshold *F_min_* = 0.5. For *k* = 7, on the other hand, the same criterion is met by *m_A_* ^(7)^ = 3 modes (see the red line), and for *k* = 13, we need *m_A_* ^(13)^ = 23 modes.

Table 1 summarizes the *m_A_* ^(*k*)^ and *m_B_* ^(*k*)^ values for all steps along the R″→T transition for *F_min_* = 0.4, 0.5, 0.6 and 0.7. Interestingly, an increasingly larger number of modes are recruited as we move away from the original equilibrium state. This important result, also confirmed for the intact complex, will be further elaborated below.

**Table 1:** *a*ANM data for the transition of a GroEL subunit between R″ and T forms(*).

Iteration (*k*)	*F_min_* = 0.4			*F_min_* = 0.5			*F_min_* = 0.6			*F_min_* = 0.7		
	*m_R″_* ^(*k*)^	*m_T_* ^(*k*)^	*RMSD* (Å)	*m_R″_* ^(*k*)^	*m_T_* ^(*k*)^	*RMSD* (Å)	*m_R″_* ^(*k*)^	*m_T_* ^(*k*)^	*RMSD* (Å)	*m_R″_* ^(*k*)^	*m_T_* ^(*k*)^	*RMSD* (Å)
0			12.33			12.33			12.33			12.33
1	1	3	11.00	1	4	10.74	1	4	10.74	2	6	10.56
2	1	3	9.77	1	3	9.57	1	4	9.46	2	4	9.16
3	1	3	8.80	1	3	8.48	1	3	8.37	2	4	7.97
4	1	3	8.01	1	3	7.61	1	4	7.37	3	4	6.96
5	1	3	7.36	1	3	6.93	3	5	6.42	5	6	6.12
6	1	3	6.77	3	4	6.16	3	4	5.70	5	7	5.29
7	3	4	6.04	3	4	5.57	5	4	5.11	6	7	4.66
8	3	4	5.46	5	4	5.09	6	4	4.53	13	7	4.14
9	3	4	5.01	6	6	4.50	11	5	4.04	23	12	3.68
10	5	4	4.58	6	7	3.98	15	6	3.60	41	16	3.27
11	6	6	4.18	13	7	3.56	23	10	3.26	73	33	2.91
12	6	7	3.73	17	7	3.21	32	16	2.96	112	44	2.59
13	10	7	3.34	23	7	2.96	64	27	2.68	210	119	2.31
14	14	7	3.06	31	16	2.70	87	39	2.43	377	259	2.06
15	17	7	2.82	54	22	2.49	156	72	2.20	576	455	1.83
16	24	12	2.62	75	35	2.30	266	169	1.99	906	727	1.63
17	32	17	2.44	111	43	2.12	422	312	1.81	1189	1118	0.87
18	52	25	2.27	164	89	1.95	651	488	1.64	-	-	-
19	72	35	2.11	267	172	1.80	953	742	1.49	-	-	-
20	98	41	1.98	385	295	1.66	-	-	-	-	-	-
21	137	68	1.86	545	442	1.53	-	-	-	-	-	-
22	192	124	1.74	769	639	1.05	-	-	-	-	-	-
23	284	207	1.64	-	-	-	-	-	-	-	-	-
24	388	304	1.54	-	-	-	-	-	-	-	-	-
25	498	443	1.44	-	-	-	-	-	-	-	-	-

(*): *m_T_* ^(*k*)^ and *m_R″_* ^(*k*)^ are the number of modes recruited at step *k*, starting from the respective states *R″* and *T*; *RMSD* values are calculated using Eq. (3). A and B designate the respective states T and R″. The first row indicates the original RMSD of 12.33 Å between the two end points.

The ***a*** **ANM** calculations thus involve two parameters, *F_min_* and *f*. The former controls the direction of motion, and the latter its size. Smaller *F_min_* values permit us to proceed via lower energy ascent directions, at the cost of longer excursions. Smaller *f* implies smaller displacements at each iteration.

(iii) The above scheme is repeated to generate a series of intermediate conformations, until the RMSD between the intermediates becomes sufficiently small (1.5 Å in Table 1). The total number of iterations, *k_tot_*, is thus defined by this targeted RMSD. The sequence of conformations along the pathway is denoted as 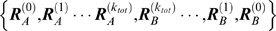 or 
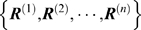
.

## Results/Discussion

### I. Allosteric transitions of a single subunit

The allosteric cycle undergone by a given subunit can be summarized as T→R→R′→R″→T. Table 2A lists the RMSDs between these alternative states, derived from the coordinates of the *cis* ring subunits in different forms of the complex. The RMSD between R′ and R″ is 1.50 Å, which is lower than the resolution of these structures, while those between different states are larger than 12 Å, except for the T and R states which differ by 5.21 Å. A reasonable approximation in view of the similarity of R′ and R″, and the resolution of existing structural data, is to condense the allosteric cycle into T→R→R″ (or R′)→T (see for example [18]).

**Table 2:** RMSD values (in Å) between (A) different forms of a subunit and (B) different states of the intact GroEL.

A. Single GroEL subunit (*)				
RMSD (Å)	1GR5_A (T)	2C7E_A (R)	2C7C_A (R′)	1GRU_A (R″)
1GR5_A (**T**)	–	5.21	12.06	12.33
2C7E_A (**R**)	5.21	–	12.23	12.35
2C7C_A (**R′**)	12.06	12.23	–	1.50
1GRU_A (**R″**)	12.33	12.35	1.50	–

B. Intact GroEL					
RMSD (Å)	1GR5 (T/T)	2C7E (R/T)	2C7C (R′/T)	1AON (R″/T)	1GRU (R″/R)
1GR5 (**T/T**)	–	6.72	12.09	11.48	12.19
2C7E (**R/T**)	6.72	–	10.85	10.65	11.03
2C7C (**R′T**)	12.09	10.85	–	3.17	2.65
1AON (**R″/T**)	11.48	10.65	3.17	–	2.67
1GRU (**R″/R**)	12.19	11.03	2.65	2.67	–

(*): based on α-carbons. The states refer to *cis* ring subunit A (indicated by suffix _A).

Toward exploring the suitability of *a*ANM for predicting the events T→R→R″→T, we first examine the *intrinsic ability* of the subunits in the T, R and R″ forms to undertake these particular changes (steps B, D and E, in Figure 1). Second, we focus on the transition R″→T, before proceeding to the intact chaperonin in section II.

#### Intrinsic dynamics of T, R and R″ states favor functional changes in structure

The lowest frequency modes, also called *global modes*, represent by definition the collective motions that are most easily accessed near a given equilibrium state, or those intrinsically favored by the 3D structure [45]. These are soft modes along entropically preferred reconfigurations [56]. We will analyze each of the three steps in T→R→R″ (or R′)→T to see to what extent the corresponding changes in structure, which are functionally required, comply with these modes.

Results are summarized in Table 3 and Figure S1. As seen in Figure S1, the structural changes induced by the slow modes allow for substantial decrease in the distance (RMSD) from the endpoint except for mode 1 for the step R→R″ (*middle* panel on the *left*) where the mode direction is apparently almost orthogonal to ***d*** ^(0)^. Table 3 lists the closest RMSDs that can be achieved upon moving along one, two, or three low frequency modes, starting from either end, or proceeding simultaneously from both ends. Strikingly, in all cases, the three modes permit the molecule to complete more than half of the structural change in the ‘functional’ direction, confirming that the subunits are intrinsically poised to reconfigure in the ‘right’ way. Below are more details. For clarity, the entries discussed below are highlighted (in boldface) in the table and more details can be found in the Supporting Information.

**Table 3:** Contributions of the lowest frequency modes to the cycle T→R→R″→T (a).

Step	Starting structure	Target structure	Original RMSD (Å)	RMSD by *the 1^st^* ANM mode			RMSD by *two* ANM modes(b)			RMSD by *three* ANM modes(b)		
				*RMSD*(Å)	*F_min_*	modes	*RMSD*(Å)	*F_min_*	modes	*RMSD*(Å)	*F_min_*	modes
**T to R**	1GR5_A (**T**)	2C7E_A (**R**)	**5.21**	**3.40**	0.57	1	**3.05**	0.65	1+2	**2.83**	0.70	1+2+3
	2C7E_A (**R**)	1GR5_A (**T**)	**5.21**	**2.83**	0.70	1	2.70	0.73	1+4	2.65	0.75	1+4+6
	Deform simultaneously		**5.21**	2.74			2.61			**2.53**		
**R to R″**	2C7E_A (**R**)	1GRU_A (**R″**)	**12.35**	12.11	0.04	1	**8.19**	0.55	1+2	7.30	0.65	1+2+3
	1GRU_A (**R″**)	2C7E_A (**R**)	**12.35**	8.84	0.49	1	7.65	0.61	1+2	7.29	0.65	1+2+3
	Deform simultaneously		**12.35**	7.57			6.02			**4.19**		
**R″ to T**	1GRU_A (**R″**)	1GR5_A (**T**)	**12.33**	**7.02**	0.68	1	6.61	0.71	1+2	6.05	0.76	1+2+5
	1GR5_A (**T**)	1GRU_A (**R″**)	**12.33**	12.03	0.05	1	8.78	0.49	1+3	7.38	0.64	1+3+4
	Deform simultaneously		**12.33**	6.49			6.19			**4.91**		

(a) The *RMSD*s refer to those between the intermediates generated by moving along the 1^st^ ANM mode (columns 5–7), two modes (8–10) and three modes (11–13) in iteration *k* = 1 (see Eq. (3)). For each step (1^st^ column), the results are separately given for the reconfiguration of the forward (first row), backward (2^nd^ row) and simultaneous (3^rd^ row) passages between the two endpoints.

(b) Lowest frequency modes that exhibit a correlation cosine of >0.1 with ***d*** ^(0)^ are selected. Note that these are all confined to the lowest frequency six ANM modes (see listed mode numbers).

- *Step T→R*. The T state exhibits a strong tendency to approach the R state. The change is indeed a reconfiguration along the first mode predicted by the ANM for the T state. This type of transition occurs both in steps A and D of the allosteric cycle (Figure 1) at the respective *cis* and *trans* ring subunits. As the T subunit changes its conformation along mode 1 alone, the RMSD from state R decreases from 5.21 to 3.40 Å, and upon further recruiting modes 2 and 3, the RMSDs decrease to 3.05 and 2.83 Å, respectively. Interestingly, the end state (R) also tends to move toward the state T, if deformed along its mode 1. These results support the view that the T form possesses an intrinsic tendency to assume its ATP-bound conformation R, prior to ATP binding, and it is also readily recovered upon nucleotide release.
- *Step R→R*″ (steps B and C in Figure 1). In this case, the RMSD of 12.35 Å between the end states cannot be reduced upon moving along mode 1. Instead mode 2 appears to play a major role, to decrease the RMSD to 8.19 Å. This step is triggered upon ATP hydrolysis, which apparently favors mode 2. Yet, it is remarkable that the contribution of three modes from both ends is sufficient to reduce the RMSD to 4.19 Å.
- *Step R*″*→T* (step E in Figure 1). Results illustrated in Table 1 already showed that the reconfiguration of R″ is initiated via a deformation along the first mode. This mode indeed yields a correlation cosine of 0.82 with the targeted direction ***d*** ^(0)^ (see Figure 3), and Table 3 shows that the original RMSD of 12.33 Å decreases to 7.02 Å upon moving along mode 1, exclusively. As to the backward transition, the first mode of the T state induces a movement almost orthogonal to ***d*** ^(0)^. Yet, recruitment of the first three modes from both ends has a dramatic effect, as an RMSD of 4.91 Å is reached.

The above analysis shows the utility of taking steps along the ANM modes so as to move efficiently toward the endpoint. Slowest modes refer here to lowest-lying eigenvalues, or softest modes. Recruiting three modes from both ends leads to intermediate states that differ by 2.53, 4.19 and 4.91 Å, as opposed to the original RMSDs of 5.21, 12.35 and 12.33 Å between the end points of the respective three steps T→R→R″→T. The extent of productive reconfiguration, or the fraction of the total path travelled (more than ½) by taking steps along these three modes is remarkable, given that (i) these represent 3/1,542 of the complete set of *3N-6* normal axes/directions that could be selected by the starting conformation, and (ii) the ANM modes are solely based on the distribution of inter-residue contacts.

#### R″→T transition analyzed by aANM: Path lengths *vs.* energy barriers

**Figure 4:**
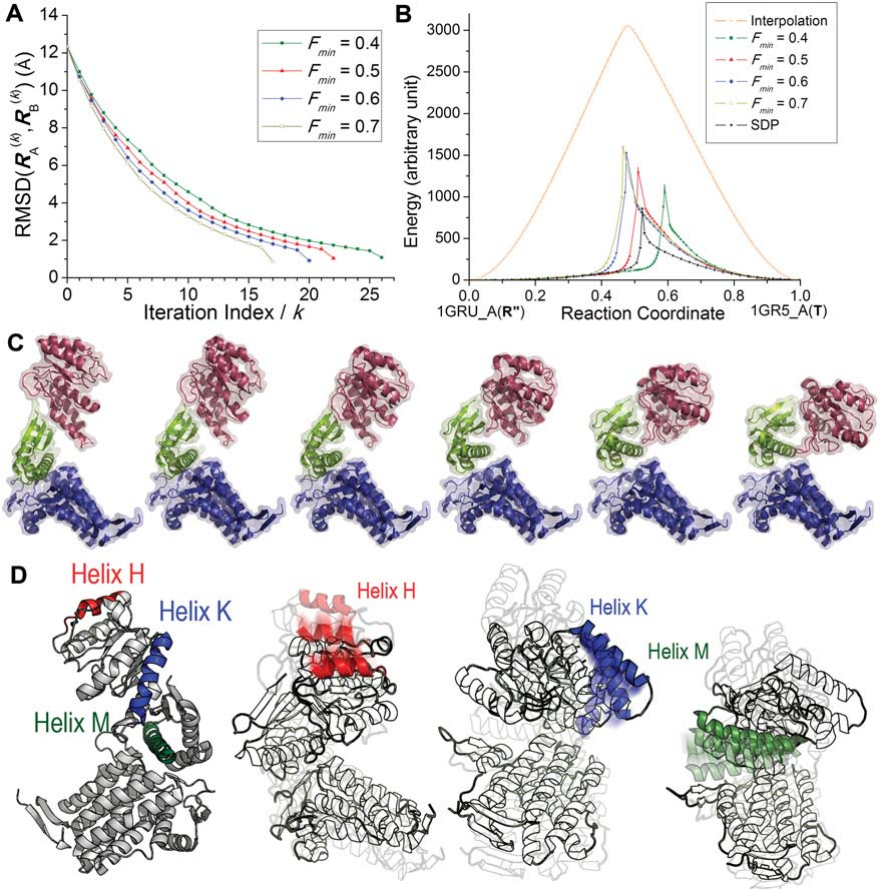
R″→T transition for a single subunit of GroEL. (A) RMSD values, |*d* ^(*k*)^| /N, between instantaneous endpoints plotted as a function of iteration number *k*. The end states refer to subunit A in the PDB structures 1GRU and 1GR5. Results are shown for *F_min_* = 0.4, 0.5, 0.6 and 0.7, corresponding to the allowable angular deviations of up to 50.8°, 45.0°, 39.2° and 33.2°, respectively, between 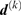 and *v* *_A_* ^(*k*)^ (or *v* *_B_* ^(*k*)^). (B) The energy profile for alternative pathways in arbitrary units. Note the significantly lower energy barrier compared to the interpolation (orange curve) between the endpoints. The black curve refers to the SDP trajectory. The reaction coordinate refers to the normalized projection of the instantaneous displacement on the original distance vector. (C) Series of conformations sampled along the reaction coordinate. The diagrams are colored by domains (equatorial, blue; intermediate, green; apical, red). (D) Movements of helices H, K and M sampled along the transition pathway. Three conformations are shown at decreasing transparency levels, starting from R″ (lightest color), to T (darkest).

Having established the use of moving along ANM modes, we now proceed to an iterative use and (re)generation of ANM modes according to the *a*ANM algorithm. In order to assess the effects of the choice of *a*ANM parameters and establish default values, which will also be adopted for the intact GroEL (see below), we repeated *a*ANM calculations for different *F_min_* values. Results for the transition R″→T are shown in Figure 4. Panel A displays the gradual decrease in the RMSD between the instantaneous pairs of intermediate conformations (see Eq.(3)) as a function of iteration number *k*, and panel B the change in energy involved in each case. It can be seen that lower *F_min_* values allow for larger excursions away from the targeted direction by recruiting relatively smaller numbers of low frequency modes. They consequently require a larger number of steps to be undertaken to reach the target, while the accompanying energy increase is relatively smaller. Higher *F_min_*, on the other hand, permits us to reach the target faster, but with a higher energy cost. The limit *F_min_* = 1 corresponds to pure interpolation by recruiting all modes.

The *reaction coordinate* in panel B is the projection of the cumulative displacement ***ν*** ^(*n*)^ = ***R*** ^(*n*)^−***R*** *_A_* ^(0)^ on the original distance vector ***d*** ^(0)^, i.e., *x*(*n*) = ***d*** ^(0)^.***ν*** ^(*n*)^/| ***d*** ^(0)^ |^2^, with R″ and T representing the respective limits *x*(*n*) = 0 and 1. The peak in the energy profile tends to be closer to the T state, especially when lower *F_min_* values (which entail lower energy barriers) are adopted. This may be related to the recruitment of higher modes (steeper ascent along the energy surface) near state T, as opposed to the first mode near R″. Note that in the allosteric transition of hemoglobin between the T and R′ states, T was found to be more predisposed to move toward R″, than R″ undergoing the reverse transition [56],[57]. In particular, the value *F_min_* = 0.5 leads to a peak around *x*(*n*) = 0.52, in accord with the steepest descent path (SDP) derived by action minimization (black curve in Figure 4B), as will be further elaborated below.

#### Sequence of events

A series of conformations visited along the transition pathway are displayed in Figure 4C for *F_min_* = 0.5. We observe that, if the E domain (blue) is fixed as a reference, the A domain (red) closes down up and twists about the axial direction, and the intermediate (I) (green) domain moves away. Helix M (386–409) on the I domain, and helices H (233–244) and K (338–354) on the A domain are highlighted in Figure 4D (*left* diagram) and shown at three successive times from different perspectives (three diagrams on the *right*) to provide a clearer description of their movements. The three color shades, from light to dark, describe three snapshots along the reaction coordinate. The movement of the I domain, and the helix M in particular, seen on the *left* panel, will be shown below to be crucial in forming/breaking critical contacts during the allosteric transitions of the intact GroEL.

An interesting observation is the sequential order of events: first the E and I domains almost stick to each other and move coherently as a single rigid body, while the A domain undergoes an upward tilting and simultaneous twisting. These movements of the A domain are completed in the first half of the transition pathway from T to R″. Then, slight rearrangements in the relative positions of the E and I domains occur, which expose the top portion of the A domain to bind the substrate and GroES. This sequence of events is consistent with the two-stage transition explored by the TMD simulations [11]. See the animations on http://www.ccbb.pitt.edu/People/yzheng/ for more details.

#### Which modes?

The number of modes involved in the transition between substates has been a question raised in a number of studies [58],[59]. Table 1 lists the number of modes, 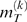 and 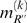, recruited at each step as the molecule travels between the end points. An important observation is the increased involvement of higher frequency modes as we proceed away from the original state. In another words, the slow modes play an important role at the initial stages of deformation, and continue to play a role throughout the entire trajectory, although they are gradually complemented by increasingly larger subsets of higher frequency modes. The dominance of low frequency modes is consistent with the previously noted driving role of large scale motions in GroEL allostery [18].

Due to the limits of applicability of the harmonic potential, it is arguable how far away from the original energy minima we can extend the quadratic approximation. In the trajectory of *F_min_* = 0.5, after 10 iterations (Table 1), the distance between the instantaneous intermediates (***d*** ^(*k*)^) falls from 12.33 Å to less than 4 Å. Only six low frequency modes out of the complete set of 1536 ANM modes accessible to the starting conformation (R″) are recruited to reach this stage, along with seven slowest modes accessible to state T. Low frequency modes are known to be robustly determined by the overall shape and contact topology of the examined structure, i.e., they are insensitive the specific interactions and nonlinear effects, hence the broad use of ENMs in NMAs in recent years [42],[60]. This property emphasized in several reviews [41],[53],[54] is also supported by the close agreement between the low frequency modes from quasiharmonic analysis of MD trajectories and those from coarse-grained NMA; see, for example, references [61] and [62]. The fact that such a large portion (up to 2/3) of the reconfiguration occurs via these robust modes lends support to the applicability of *a*ANM. A small portion near the PTS needs, however, to be interpreted with care, as will be further analyzed below.

In a strict sense, the normal modes provide information on the direction of motion near an energy minimum, and steps along these modes will be accurate to the extent that they are infinitesimally small. In order to examine how the step size *s_A_* ^(*k*)^ (or *s_B_* ^(*k*)^) affects the course of structural reconfiguration, we repeated our calculations with various scaling factors *f* in the range 0.1<*f*<1. As described above *f* = 1 allows for taking the full step size that minimizes ***d*** ^(*k*)^ at a given iteration *k*, but entails possible unphysical distortions in the structure. Calculations with different *f* values showed that the parameter *f* = 0.2 can be safely adopted to avoid unrealistic deformations in backbone geometry. In particular, we monitored the C^α^-positions and C^α^-C^α^ bond lengths, which refer to bonded and non-bonded interactions in the coarse-grained model used here, to verify that the conservative steps generated with this scaling factor avoid steric clashes between residue pairs and unrealistic fluctuations in bond lengths. See more details (Figure S3 and Table S1) in the Supporting Information.

#### Comparison with the results independently obtained by action minimization (SDP)

Toward assessing the consistency of our results with those predicted by other analytical methods, we examined the transition pathway predicted for the same model by the action minimization algorithm [29],[63]. The resulting trajectory, called the steepest descent pathway (SDP), led to the energy profile shown by the black curve in Figure 4B. The barrier height is slightly lower than that obtained by *a*ANM, while the shape of the energy profile shows close resemblance: a peak around *x*(*n*) = ∼0.52, preceded by minimal energy cost for an extended portion of the reaction coordinate, followed by a sharp increase near the peak, and then a smooth decrease after the peak.

**Figure 5:**
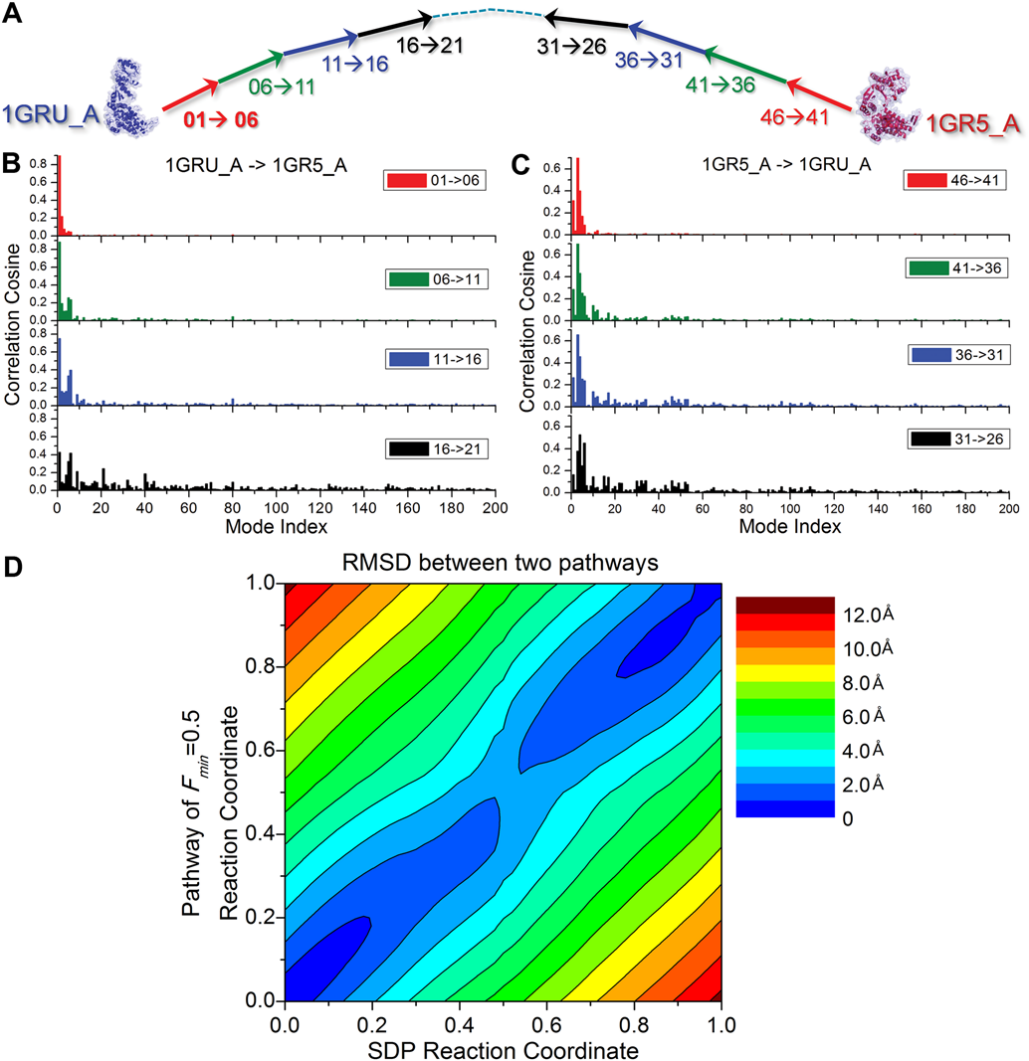
Comparison with the results from steepest descent pathway (SDP) based on action minimization. (A) Fragmentation of the SDP pathway for the transition 1GRU←→1GR5 of a subunit into nine macrosteps, consisting each of five frames. Same color scheme is adopted in panels B and C. (B) Correlation between SDP macrosteps and ANM modes accessible to the original conformation 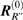. (C) Same as panel B, for the right portion of the trajectory, i.e. the reconfiguration from 1GR5_A to 1GRU_A using the eigenvectors 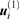 generated for 1GRU_A. Note that the early macrosteps from both directions are accounted for by a few slowest ANM modes, while increasingly higher modes are being recruited as the molecule proceeds away from its original conformation, consistent with the results found by *a*ANM (see Table 1). (D) RMSD values between the intermediate conformations sampled by the *a*ANM and SDP methods. The *a*ANM results refer to the trajectory *F_min_* = 0.5. The RMSDs between pairs of intermediates remain lower than 2.0 Å at all steps (see the color-coded scale on the right).

Toward a more critical analysis of the modes that contribute to the SDP, we reorganized the SDP trajectory (consisting of 46 frames) into a series of *k = 9* (*macro*)*steps* by collapsing each set of five consecutive frames into a macrostep (Figure 5A) and we calculated the deformation vector Δ***R*** *_k_* ^SDP^ = ***R*** *_n+5_* ^SDP^−***R*** *_n_* ^SDP^ for each macrostep. The following questions were raised: Which ANM modes effectively contribute to these macrosteps? Do SDP macrosteps exhibit the same tendency as *a*ANM to originally proceed via softer modes and gradually recruit increasingly larger subsets of modes? How similar are the conformations visited along the *a*ANM and the SDP? To this aim, we evaluated the correlation cosine between Δ***R*** *_k_* ^SDP^ and the ANM modes ***u*** *_iA_* ^(1)^ and ***u*** *_iB_* ^(1)^ accessible to original states ***R*** *_A_* ^(0)^ and ***R*** *_B_* ^(0)^. The results are shown as a function of mode index *i* in the respective panels B and C of Figure 5. The correlation cosines represent the relative contributions of the intrinsically accessible ANM modes to the SDP macrosteps. In accord with the results from *a*ANM, only very few modes at the low frequency end of the spectrum contribute to the SDP macrosteps in the close neighborhood of the original states (red plots). The slow modes contribute by almost by the same amount as those observed in *a*ANM at the successive stages of the transition pathway. The contribution of higher frequency modes, which is negligibly small at early stages, gradually increases, consistent with the *a*ANM.

An even more direct comparison of the conformations sampled by the *a*ANM and the SDP is provided by the map in Figure 5D. The map displays the RMSDs between the conformations sampled by the SDP at successive steps (abscissa) and those sampled by the *a*ANM algorithm (ordinate) using *F_min_* = 0.5. The RMSDs remain lower than 2 Å for the most part of the trajectory.

### II. Allosteric transitions of the intact chaperonin

#### Intrinsic dynamics of the T/T, R/T, R′/T and R″/R states

Calculations performed above for a single subunit demonstrated that the transition pathways between the functional forms of the single subunit can be delineated by the *a*ANM algorithm. In particular, Table 3 and Figure S1 showed that a few low frequency modes can account for a significant portion of the structural changes that take place between the functional forms. We now explore the allosteric transitions in the intact chaperonin. We begin by examining if/how the low frequency modes play equally an important role in the allosteric dynamics of the intact GroEL.

The counterparts of Table 3 and Figure S1 for the intact chaperonin are Table 4 and Figure S2. Here, the major difference is the fact that the changes occur via non-degenerate modes that maintain the heptameric symmetry of the GroEL structure, similar to the previously noted [64],[65] dominant role of non-degenerate ANM modes in enabling the maturation of icosahedrally symmetric viral capsids. Results are listed for the first 1, 3 and 6 non-degenerate modes. Note that the set of ANM modes accessible to the GroEL complex is larger than that of the single subunit by a factor of 14. We might thus expect to recruit a larger number of modes in the low frequency regime to achieve the same fractional contribution to observed changes. However, it is again striking to observe that six non-degenerate modes are sufficient to generate intermediates that are almost half way through the transition between the end points. The original RMSD values of 6.72, 10.85 and 11.54 Å between the end states of the three respective steps T/T→R/T, R/T→R′/T and R″/R→T/R are reduced to 3.26, 6.35 and 7.87 Å upon moving along these modes (see Table 4). The gradual decreases in the RMSDs as the intact chaperonin reconfigures along these modes are shown in Figure S2.

**Table 4:** The contribution of lowest frequency modes to the three major steps of the chaperonin allosteric cycle(a).

Step	Starting structure	Target structure	Original RMSD (Å)	RMSD by the *1^st^* non-degenerate mode			RMSD by the *first 3* non-degenerate modes			RMSD by the *first 6* non-degenerate mode		
				*RMSD*(Å)	*F_min_*	modes	*RMSD*(Å)	*F_min_*	modes	*RMSD*(Å)	*F_min_*	modes
**T/T to R/T**	1GR5	2C7E	**6.72**	**5.54**	0.32	1	4.91	0.46	1+10+11	3.97	0.65	1+10+11+28+51+61
	2C7E	1GR5	**6.72**	5.83	0.24	3	4.66	0.52	3+12+20	3.42	0.77	3+12+20+35+49+56
	Deform simultaneously		**6.72**	5.54			4.65			**3.26**		
**R/T to R′/T**	2C7E	2C7C	**10.85**	10.34	0.09	3	9.96	0.15	3+17+20	6.35	0.66	3+17+20+29+44+49
	2C7C	2C7E	**10.85**	10.68	0.03	3	9.96	0.16	3+4+10	9.34	0.27	3+4+10+20+21+22
	Deform simultaneously		**10.85**	10.34			9.77			**6.35**		
**R″/R to T/R**	1GRU	2C7E′ (b)	**11.54**	11.22	0.05	3	9.35	0.34	3+10+17	8.34	0.48	3+10+17+20+22+30
	2C7E′	1GRU	**11.54**	11.19	0.06	3	9.33	0.35	3+20+27	8.52	0.46	3+20+27+29+32+35
	Deform simultaneously		**11.54**	11.19			8.43			**7.87**		

(a) Major steps are those involving an RMSD larger than 3.2 Å (∼resolution of some structures) between the end points.

(b) 2C7E′ is same as the PDB structure 2C7E, except for the inversion shown in step F of the allosteric cycle in Figure 1.

#### Energy profile and operating modes

**Figure 6:**
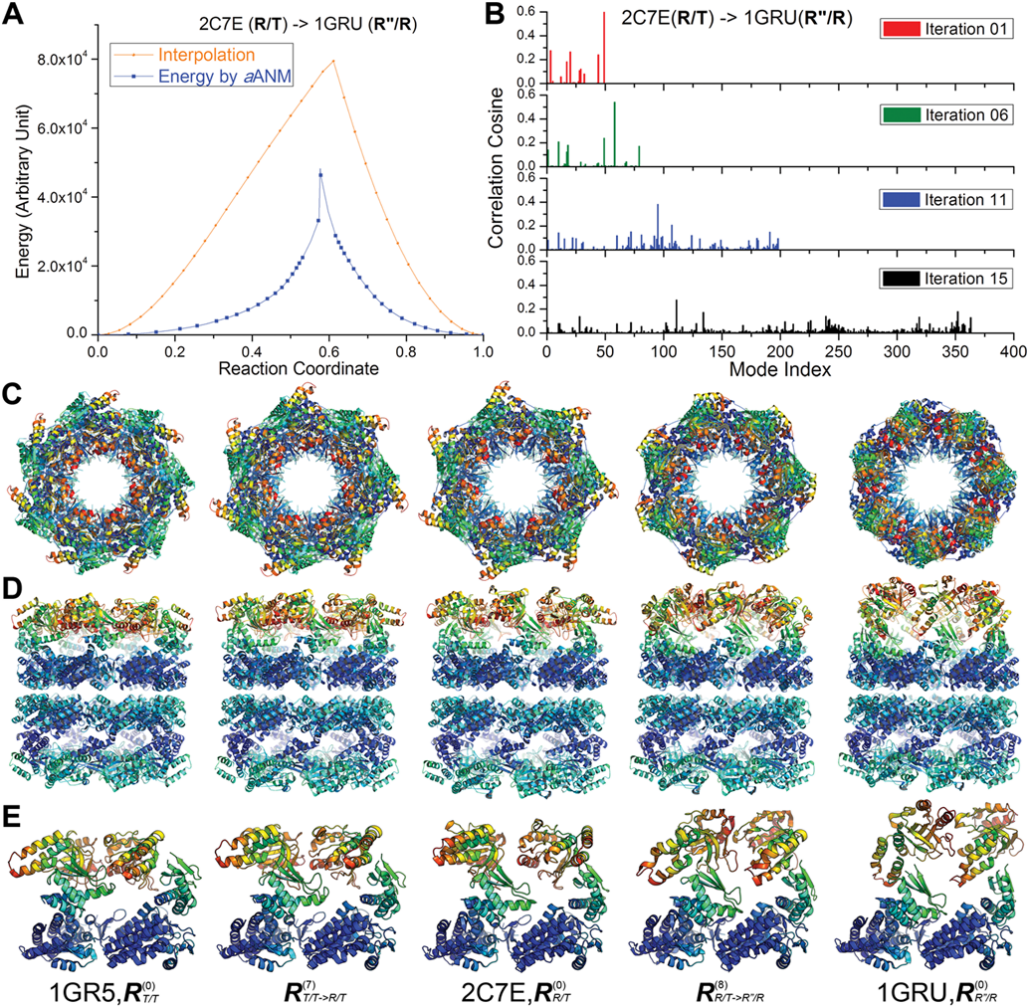
Transition among T/T→R/T→R″/R forms of the intact GroEL complex. (A) Energy profiles of the intact GroEL complex (R/T→R″/R) along the reaction coordinate computed by *a*ANM (blue) and Cartesian interpolation (orange) using the double-well potential given by Eq. (7). (B) Contribution of different modes at various steps (1, 6, 11 and 15) along the transition R/T→R″/R. Broader numbers of higher frequency modes are recruited as the structure approaches the energy barrier (see Table 5). (C) Top view of structures sampled along the transition. Snapshots corresponding to conformations 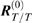, 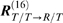, 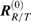, 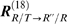 and 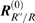 are shown. (D) Side view of the same structures. (E) Close-up views of pairs of adjacent subunits. The diagrams in panels C–E are color-coded according to the mobilities of residues (red: most mobile; blue: almost fixed). Note that the equatorial domains of *cis* ring subunits are almost fixed, while the largest motions occur at the apical domains of the same subunits.

We now proceed to the *a*ANM study of the transition T/T→R/T→R″/R. These three states are selected because they differ by an RMSD of 6.72 Å and 11.03 Å, respectively (see Table 2 part B). R′/T and R″/T differ from R″/R by less than 3 Å, which is comparable to the resolution of the structures, hence their exclusion from the above scheme. The results are presented in Figure 6 and Table 5. The energy profile along the transition R/T→R″/R (Figure 6A) exhibits a shape similar to that obtained with the single subunit; the PTS is closer to the R″/R state (*x*(*n*) = 0.58), and the barrier is much lower than that encountered upon interpolation (orange curve) between the end points.

**Table 5:** *a*ANM data for the transition of Intact GroEL complex(*).

*k*	1GR5 (T/T) to 2C7E (R/T)			2C7E (R/T) To 1GRU (R″/R)		
	*m*_T/T_^(*k*)^	*m*_R/T_^(*k*)^	*RMSD* (Å)	*m*_R/T_^(*k*)^	*m*_R″/R_^(*k*)^	*RMSD* (Å)
0	–	–	6.72	–	–	11.03
1	51	20	6.17	49	121	9.70
2	51	35	5.67	49	122	8.53
3	61	51	5.04	49	139	7.58
4	89	51	4.55	49	142	6.86
5	94	65	4.11	54	181	6.14
6	118	104	3.74	79	188	5.58
7	218	118	3.40	120	189	5.07
8	238	183	3.10	95	209	4.61
9	282	205	2.80	86	310	4.21
10	326	229	2.55	142	338	3.80
11	425	282	2.32	198	384	3.44
12	515	346	2.14	240	471	3.10
13	620	510	1.95	318	576	2.80
14	942	616	1.80	321	731	2.53
15	-	-	-	363	1054	2.31
16	-	-	-	519	1380	2.13
17	–	–	–	636	1869	1.93
18	–	–	–	792	2281	1.23

(*): *k* = 0 refers to the original RMSD between the end points. Results are obtained with *F_min_* = 0.5.

Figure 6B displays the histograms of modes contributing at various iterations (*k* = 1, 6, 11 and 15), starting from the R/T state. The tendency to recruit higher modes as the PTS is approached is clearly seen, consistent with the trend observed in the single subunit with both *a*ANM and SDP. Table 5 shows that the RMSD between the intermediate conformations reduces to 1.93 Å by selecting modes from amongst the slowest 636 modes. More detailed examination shows that among them 97 non-degenerate modes contribute to 90% of the structural change. This subset amounts to less than 0.5% of the spectrum of ANM modes (∼2.2×10^4^ of them).

Panels C, D and E display the evolution of the intact structure viewed from the top (C), and from the side (D), along with a close-up view of two adjacent subunits on the *cis* ring (E). See http://www.ccbb.pitt.edu/People/yzheng/ for movies. The *trans* ring is generally observed to undergo moderate changes between the T to R states. The *cis* ring, on the other hand, undergoes concerted rotations and extensions at the apical and intermediate domains. Notably, the intermediate domains move toward the cleft between neighboring equatorial domains, while the apical domains extend along the vertical (cylindrical axis) direction accompanied by a rotation about the same axis. These motions result in the enlargement of the central cavity along with the exposure of the apical domains on the *cis* ring to bind the GroES-binding flexible loop K15-T36.

#### Redistribution of inter-subunit salt bridges and implications on intra-ring cooperativity

Salt bridges at the interface between the *cis* and *trans* rings have been pointed out in site-directed mutagenesis experiments by Saibil and coworkers to play a critical role in communicating allosteric signals between the two rings and the co-chaperonin binding site [12]. The redistribution of critical interactions accompanying the global changes in structure has been proposed to be a possible mechanism for regulating allosteric signal transduction [66]. Here we examine more closely the changes in the distances between salt-bridge forming residues during the transitions of the intact chaperonin, and discuss our results in relation to previous experimental and computational data.

**Figure 7:**
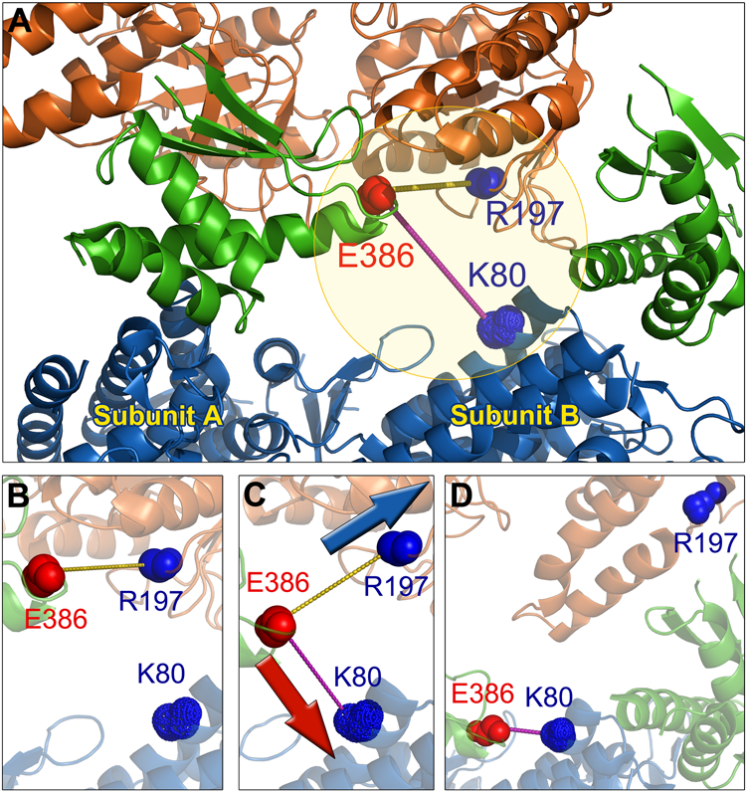
The *cis* ring inter-subunit interactions during the transition T→R″, based on the intact GroEL structure calculation. (A) Intersubunit interface near the intermediate domains (green) of two adjacent subunits in the *cis* ring. The backbones are shown in cartoon view and colored by domains: A (orange), I (green), and E (blue). Backbone atoms of three charged residues are shown by spheres. Positively and negatively charged residues are colored blue and red, respectively. (B) The inter-subunit hydrogen bond, E386-R197, in the T state of the *cis* ring (1GR5). (C) During the transition to state R″/R, residue E386 in the I domain moves towards K80 (blue sphere) in the E domain of the adjacent subunit, while R197 on the A domain moves away from E386. (D) The final configuration in the R″ state of the *cis* ring, represented by 1GRU. Residue E386 now forms a new hydrogen bond with K80.

Experiments conducted with Arg197Ala mutant [67] pointed to the functional role of the salt bridge between E386 and R197 on adjacent subunits in the *cis* ring. This inter-subunit salt-bridge was also noted in early cryo-EM studies [68]. It forms in the T state of the ring (1GR5) (Figure 7A and 7B), and it has been proposed to be an essential component of the positive intra-ring cooperativity [69]. The R″ state of the same ring (1GRU) indicates, on the other hand, a new salt bridge, formed between E386 and K80 (on the E domain of the neighboring subunit) (Figure 7D). The *a*ANM results shed light to the mechanism of this interchange of salt bridges. The separation between E386 and R197 α-carbons, originally equal to 15.7 Å (their charged ends being separated by 3.2 Å), gradually increases by the downwards motion of the intermediate domain. After 18 iterations (for T/T→R/T), the distance between E386 and R197 becomes larger than that between E386 and K80, and K80 replaces R197 to form a salt bridge with E386 (Figure 7C). Afterwards, R197 moves dramatically away from E386, led by the opening of the apical domain. Meanwhile the downward movement of helix M continues until the distance between the C^α^-atoms of the new salt-bridge-forming residues E386 and K80 reduces to 8.6 Å.

These intra-ring rearrangements are in accord with previous TMD simulations [11]. However, TMD runs showed heterogeneous subunit behavior, while the *a*ANM trajectory retains the sevenfold rotational symmetry. In each ring, the movements of all subunits are identical and coherent (Figure 6C). In Figure 6B we noted that not all slow modes in the lower end of the spectrum contribute to the transition. Instead a subset inducing cylindrically symmetric changes is recruited. For example, in the first iteration, while 51 modes are recruited, the contributions of modes 2 through 9 are almost zero, and we have large contributions from the non-degenerate modes 1, 10, 11, 28 and 51 (Table 4). Our results support the Monod-Wyman-Changeux (MWC) view [70],[71] of cooperative, symmetric (non-degenerate) modes of motion controlling allostery. Likewise, GroEL engineering experiments provide evidence for the concerted nature of the allosteric transition [69].

#### Comparison with the results from recent BD simulations

A recent examination of the time evolution of salt-bridges formed/broken at the steps T→R and R→R″ of the GroEL allosteric cycle brought attention to a number of key interactions [18]. Those results have been obtained by Hyeon, Lorimer and Thirumalai, by performing BD simulations, for a dimer, using state-specific Go-like potentials for the end points [18]. Therefore, they differ from ours in their method, model and parameters. Yet, in order to see if similar trends could be detected in spite of the differences in the methodology, we undertook a careful examination of the formation/breakage of salt bridges along the *a*ANM predicted reaction coordinates.

**Figure 8:**
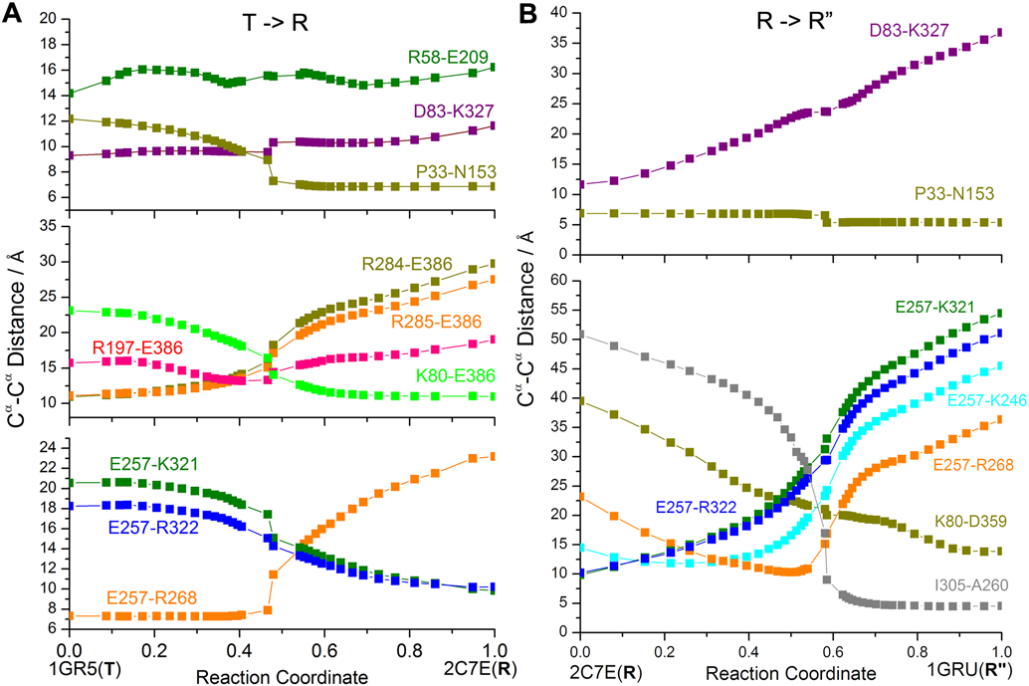
Changes in the distances between salt-bridge forming pairs along the *a*ANM reaction coordinate. Results are shown for (A) T→R and (B) R→R″ transitions. See Figure S4 for the corresponding time dependences, and Table 6 for the kinetic expressions and the comparison with the results from BD simulations by Hyeon *et al.* [18].

Figure 8 displays our results for the respective T→R (panel A) and R→R″ (panel B) transitions of *cis* ring subunits obtained by aANM for the intact chaperonin. These are the C^α^-C^α^ distances between intra- or intersubunit salt-bridge forming residues, plotted as a function of reaction coordinate. Interestingly, particular pairs or residues exhibit a smooth increase/decrease in their distance, whereas others are more sluggish. The former group of pairs which readily associate/dissociate may be viewed as driving interactions. We notice, for example, the smooth decrease in K80-D359 distance along with increase in D83-K327 distance (panel B), indicative of the compensating formation and breakage of these two intra-subunit salt bridges prompted at an early stage of the R→R″ transition. These changes undergone early on are in sharp contrast to the salt bridges involving E257 and E386, which exhibit strong resistance to dissociate or associate (panels A and B). The leading role of K80-D359 and D83-K327 is in agreement with the mechanism observed in BD simulations [18]. The formation of the K80-D359 salt-bridge has been pointed out therein to be the major driving force for the R→R″ transition [18]. Also, this formation has been pointed out to be coupled to the dissociation of D83-K327 [18]. Other similar features include the fast increase in R58-E209 and decrease in P33-N153 distances at short times during T→R, in contrast to the slower responses of D83-K327 and E257-R268 at the same stages (panel A), succeeded by the high stability of P33-N153 during R→R″, the resistance of E257-K321 and E257-R322 to come closer at the initiation of T→R, in contrast to their predisposition to dissociate at the onset of R→R″. Finally, it is interesting to notice that the non-monotonic behavior of the pair E257-R268 in panel B conforms to previously observed [18] ensemble-averaged behavior for the same pair; while that of R197-E386 observed here departs from the smooth increase previously reported.

In the interest of making an even more stringent, quantitative comparison between the two sets of data, we have considered the relative rates of formation/rupture of different salt bridges. Our approach does not contain ‘time’ explicitly. Instead we have the ‘number of iterations’. In order to evaluate the relative time dependence of the salt bridge formation/breakage events, we normalized one of our curves, called ‘reference curve’, with respect to its counterpart obtained [18] by Hyeon *et al.* (2006). This permitted us to plot the accompanying time dependence of all other salt bridges as a function of time (see Figure S4) and determine the best fitting kinetic data. The results, compiled in Table 6 exhibit striking quantitative agreements between the *a*ANM results and those from BD simulations.

**Table 6:** Comparison of the kinetics of salt-bridge forming residues obtained by *a*ANM and BD simulations(a).

Transition	Salt Bridge	Time dependence of C^α^-C^α^ distance ( *a*ANM) (b)	R^2^ (*a*ANM)	<d(t)> (from reference [18])	Correlation coefficient (c)
**T→R**	D83-K327	11.8−2.3*e* ^−t/101.1^	0.91	13.4−4.9 *e* ^−t/100.0^	0.999
	P33-N153	6.8+5.8 *e* ^-t/7.7^	0.99	7.3+4.2*e* ^−t/6.3^	0.998
	R284-E386	30.7−9.9*e* ^−t/15.8^−10.4*e* ^−t/84.2^	0.99	29.7−8.1*e* ^−t/20.8^−8.4*e* ^−t/85.8^	0.999
	R285-E386	Reference curve	–	28.4−6.6*e* ^−t/19.1^−8.1*e* ^−t/88.8^	1
	R197-E386	22.4−4.1*e* ^−t/20.0^−6.4 *e* ^−t/307.2^	0.99	20.9−2.6*e* ^−t/0.67^−6.4*e* ^−t/96.7^	0.975
	K80-E386	11.0+10.1*e* ^−t/14.1^+4.0*e* ^−t/2.5^	0.99	10.4+7.6*e* ^−t/12.1^+2.2*e* ^−t/61.8^	0.993
	E257-R268	22.5−169.2*e* ^−t/58.3^+151.9*e* ^−t/62.0^	0.99	21.8−4.2*e* ^−t/26.2^−7.9*e* ^−t/66.4^	0.998
	E257-R322	10.3+8.1*e* ^−t/24.8^	0.99	N/A	–
	E257-K321	10.1+10.7*e* ^−t/26.6^	0.99	N/A	–
**R→R″**	D83-K327	Reference curve	–	37.3−26.9 *e* ^−t/77.9^	1
	E257-K321	58.1−55.1*e* ^−t/73.1^	0.94	N/A	–
	E257-R322	54.6−50.7*e* ^−t/76.2^	0.94	N/A	–
	E257-K246	51.6−47.6 *e* ^−t/95.8^	0.86	N/A	–
	K80-D359	15.1+25.2*e* ^−t/37.4^	0.98	14.1+26.4*e* ^−t/28.0^	0.991

(a) BD simulations results were reported by Hyeon *et al.* (2006) [18]; time (t) in microseconds.

(b) *a*ANM results are reported for all salt-bridges that exhibited a monotonic time dependence (single or double exponential) with R^2^>0.85.

(c) between the two sets of data (*a*ANM and BD) for each salt bridge.

#### Critical interactions formed/broken at the transition involve conserved residues

The above results (Figure 8) reveal that certain interactions play a key role in stabilizing particular states, once formed. For example, the inter-subunit E257-R268 salt bridge appears to be crucial for the T state of the *cis* ring; whereas the inter-subunit hydrophobic contact I305-A260 seems critical to stabilizing the R″ state. These interactions are usually disrupted only at the PTS.

Toward a more systematic assessment of such critical interactions, we focused on the changes in native contacts occurring at the PTSs. A set of 2028 native contacts are shared between the R and R″ states (native contacts being defined as C^α^-C^α^ distances lower than 7 Å, in the present model) while the two respective states exhibit 137 and 105 distinctive native contacts. The questions are: How does the number of native contacts vary along the transition pathway? Which contacts among those differentiating the two end structures are maintained along the trajectory and which ones are broken or formed only at the transition state?

**Figure 9:**
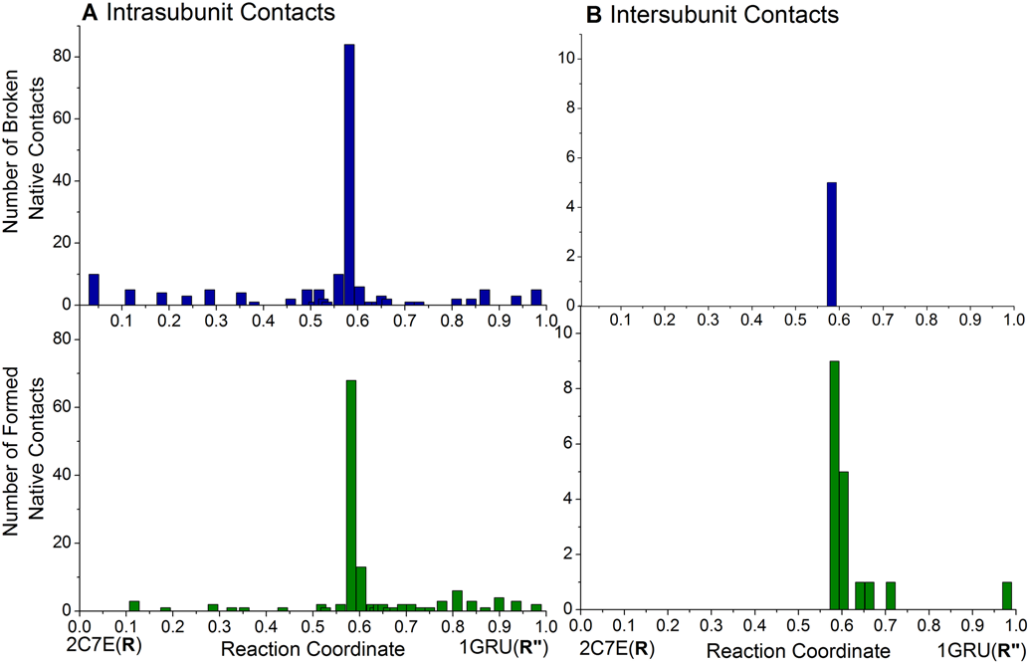
Evolution native contacts along the structural transition from R to R″ states. The number of intra-subunit (panel A) and inter-subunit (panel B) native contacts that are disrupted (upper panel) and formed (lower panel) *vs.* the reaction coordinate. The results refer to *F_min_* = 0.5 for *cis* ring subunits along the transition from 2C7E (R/T) to 1GRU (R″/R). Each bar represents the number of native contacts formed/broken at a given *a*ANM iteration. Note the sharp increase near the energy barrier. See Figure 10 for the corresponding critical contacts.

Figure 9A displays the time evolution of native contacts observed for a single subunit during the transition R→R″; and panel B shows its counterpart for inter-subunit contacts observed in the complex. Clearly, most of the native contacts remain unchanged throughout a large portion of the trajectory, while significant changes occur near the PTS, both with regard to the disruption (*top* panel) and formation (*bottom* panel) of contacts. Trajectories generated by varying *a*ANM model parameters in the ranges 0.4<*F_min_*≤0.7 and 0.2≤*F_min_*≤0.5 yielded almost identical results, confirming the strong preference of the molecule to redistribute native contacts only in the vicinity of the PTS, while the large portions of the trajectories are enabled by minimal changes in native contacts. See also Figure S4 in the Supporting Information for the step R″→T.

**Figure 10:**
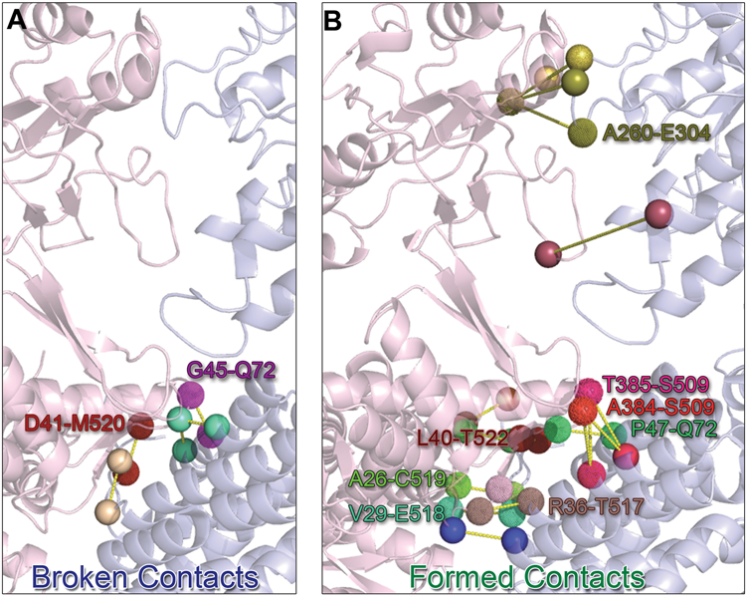
Redistribution of inter-residue contacts at the transition from R to R″ state. Panel (A) shows the inter-residue contacts between adjacent subunits of the *cis* ring, which break up during the transition. The two subunits are colored in light pink and blue. Contact pairs are represented by spheres at the C^α^ position, and by distinctive colors. Similarly, panel (B) shows the contacts newly formed during the transition. See Table 7 for the complete list of residue pairs shown here. The contacts involving a pair of conserved residues are labeled using the same colors as the corresponding residues.

A closer examination reveals the residues involved in critical interactions tend to be strongly, if not fully, conserved. Table 7 lists the inter-subunit residue pairs broken/formed at the transition, also called ‘critical intra-ring contacts’, along with their conservation scores evaluated using ConSurf [72],[73]. The E domains make indeed many more inter-subunit contacts than the A domains (Figure 10). As a result the A domains can move independently of one another, while the E domains are constrained [18], which explains their breakage at the critical state only. It is also interesting to note that the only I domain residues that make critical contacts (Ala384 and Thr385 next to the N-terminus of the M helix) undergo large reorientations (Figure 4D). We also note the segment A384-V387 that has been pointed to play an important role in mediating positive intra-ring cooperativity [16].

**Table 7:** Critical inter-subunit contacts broken/formed during R→R″.

	Chain A		ConSurf score(b)	Chain B		ConSurf score(b)
	Residue type	Index(a)		Residue type	Index(a)	
**Breaking**	VAL	39 (E1)	0	**GLU**	**518** (E2)	9
	**ASP**	**41** (E1)	9	**MET**	**520** (E2)	9
	**GLY**	**45** (E1)	9	**GLN**	**72** (E1)	9
	ALA	46 (E1)	0	**MET**	**69** (E1)	9
	ALA	46 (E1)	0	**GLU**	**76** (E1)	9
**Forming**	**ALA**	**26** (E1)	9	**CYS**	**519** (E2)	9
	**VAL**	**29** (E1)	9	**GLU**	**518** (E2)	9
	LYS	34 (E1)	0	MET	114 (E1)	9
	**ARG**	**36** (E1)	9	**THR**	**517** (E2)	9
	**ASN**	**37** (E1)	9	**CYS**	**519** (E2)	9
	**LEU**	**40** (E1)	9	**THR**	**522** (E2)	9
	**PRO**	**47** (E1)	9	**GLN**	**72** (E1)	9
	ILE	60 (E1)	7	**LYS**	**4** (E1)	9
	**GLU**	**61** (E1)	9	ALA	2 (E1)	0
	THR	210 (A)	1	GLN	351 (A)	0
	**ALA**	**260** (A)	9	**GLU**	**304** (A)	9
	**ALA**	**260** (A)	9	ILE	305 (A)	0
	**ALA**	**260** (A)	9	GLY	306 (A)	0
	**VAL**	**264** (A)	9	GLY	306 (A)	0
	**ALA**	**384** (I2)	9	TYR	506 (E2)	0
	**ALA**	**384** (I2)	9	**SER**	**509** (E2)	9
	**THR**	**385** (I2)	9	TYR	506 (E2)	0
	**THR**	**385** (I2)	9	**SER**	**509** (E2)	9

(a) Conserved residues are highlighted in boldface. The domains are shown in parentheses. The equatorial domain residues are Met1-Pro137 (E1) and Val411-Pro525 (E2); intermediate domain residues are Cys138-Gly192 (I1) and Val376-Gly419(I2); and the apical domain (A) consists of a contiguous segment Met193-Gly375.

(b) The scores are based ConSurf Server [72],[73] calculation for GroEL sequence.

Broken pairs distinguished by their high conservation are Asp41-Met520 and Gly45-Gln72; and those conserved contacts made at the PTS are Ala26-Cys519, Val29-Glu518, Arg36-Thr517, Asn37-Cys519, Leu40-Thr522, Pro47-Gln72, Ala260-Glu304, Ala384-Ser509 and Thr385-Ser509 (Figure 10). Notably, some of these residues have been already reported to be functional. The mutation Gly45Glu, for example, causes defective release of partial folded polypeptide without reducing ATPase or GroES binding [74],[75]. Val264Ser reduces GroES and polypeptide binding affinity [74]; Ala260 is involved in binding and unfolding rhodanese, as shown in MD simulations [76]. Mutants Arg36His, Glu76Lys, Val264Ile, Thr385Ile, Met520Ile and Thr522Ile give rise to active single-ring GroEL [77]. Finally, we note that all four residues, Val39, Asp41, Gly45 and Ala46, listed in Table 7 to break at the energy barrier, belong to the segment Val38-Ile49, pointed out in our previous study [16] to play a key role in establishing the intra-ring allosteric communication.

### Conclusion

GroEL is a molecular machine that has been broadly studied in recent years using both experimental and theoretical or computational methods. Yet, a structure-based analysis of the transition of the intact chaperonin between its functional forms has been held back by the large size of the chaperonin. The *a*ANM method is proposed as a first approximation toward approaching this challenging task.

The conformational transition results from the competition of two counter effects at a minimum, one intramolecular, and the other intermolecular: (1) any deformation away from the original equilibrium states involves an uphill motion along the energy surface, but the lowest frequency motions incur the lowest ascent in energy (intramolecular); (2) longer pathways experience higher frictional resistance from the surroundings, such that the shortest pathway may be favored (intermolecular) at the expense of internal strains. In the *a*ANM model, one can balance these two counter effects by adopting different threshold correlation cosine (*F_min_*), for different proteins in various conditions. Higher *F_min_* values emphasize the effect (2), with the upper limit *F_min_* = 1 restricting the pathway to a pure interpolation. Lower *F_min_* values on the other hand allow for occasional departures from the targeted direction, with the limit *F_min_* = 0 tolerating steps orthogonal to the target direction.

Central to the development of this methodology were approximations and parameters that needed to be critically tested, hence our extensive examination of the transitions of a GroEL subunit and comparison with the results from other methods, including SDP (action minimization), MD simulations [11] and experimental studies. Multiple pathways were explored to this aim by varying the parameters *F_mi_*_n_ and *f*, which control the respective direction and size of the *a*ANM steps. It is conceivable that multiple routes are accessible between the two endpoints, given the multidimensional character of the energy landscape. For example, broad transition state ensembles have been observed by Hyeon, Lorimer and Thirumalai in their simulations of the GroEL allosteric transitions using a self-organized polymer model [18]. However, one or more are more probable, and *a*ANM method by definition aims at sampling such probable (structurally favored) paths. Of interest was to identify the common features, if any, of the paths sampled with different parameters, and to identify among them those most closely agreeing with the data from other methods and experiments. Notably, the generated pathways invariably exhibited the following common features, observed for a single subunit, and confirmed in the intact chaperonin:

- The recruitment of low frequency modes near the original state, succeeded by the recruitment of higher frequency modes near the barrier (see for example Table 1 and Figures 3, 5A–C, and 6B),
- The dominant role of a few well-defined low frequency modes for achieving substantial (at least halfway) displacement in the ‘functional’ direction (Tables 2 and 4 and Figures S1 and S2),
- The close agreement between pathways and energy profiles generated with different parameters, observed over a large portion (*x*(*n*)≤0.4 and *x*(*n*)≥0.7) of the reaction coordinate, also in accord with SDP results (see for example Figures 4A and 4B, 5D, and S6), consistent with the predominance of well-defined modes at the initial stages of the structural changes,
- Barrier heights significantly lower than that incurred upon linear interpolation of coordinates between the two endpoints (Figures 3B, 5A, and S6),
- Protection of the native state inter-residue contacts throughout a surprisingly large portion of the transition pathway, and formation/breakage of native contacts, mostly at the E domains, only in the close neighborhood of the PTS (Figures 10 and S5 and Table 7).

Having established these common features, a closer analysis of intermediate conformations, energetics and mode distributions in comparison to those from the rigorous SDP method supported the use of *F_min_* = 0.5 as an optimal parameter for further calculations for the intact chaperonin. A discriminative feature that supported this parameter was the position of the energy barrier along the reaction coordinate, in accord with SDP. The close neighborhood of the energy barrier is indeed the region most sensitive to the choice of parameters. Notably, this region involves local rearrangements (RMSD<2 Å) comparable (or below) the resolution of examined structures. Therefore the results in this regime should be interpreted with caution. However, the set of ‘critical’ contacts identified for the R→R″ transition (listed in Table 7) have been verified to be robustly reproduced over a relatively broad range of *a*ANM parameters (0.4<*F_min_*≤0.7 and 0.2≤*f*≤0.5). Another observation in strong support of *a*ANM results with *F_min_* = 0.5 is the striking qualitative and quantitative agreement between the dynamics of salt-bridges observed here and those found by BD simulations with state-dependent energy functions and parameters [18], presented in Figures 8 and S4, and Table 6.

The application of *a*ANM to GroEL therefore elucidates not only highly probable pathways or the hierarchic contribution of modes to achieve the transition; but it also provides insights into key interactions that initiate the transition (e.g., formation of the K80-D359 salt-bridge), or those that form/break at the transition state(s). Such inter-subunit contacts disrupted and formed at the PTS are illustrated at the respective panels A and B of Figure 10. Notably, the majority of the residues involved in these critical interactions are highly conserved (Table 7), and some of them have been observed in previous site-directed mutagenesis experiments to affect GroEL machinery [74]–[77]. The importance of the other identified critical contacts to the functional transitions of the chaperonin remains to be further established by experiments.

On a practical side, the major utility of the method lies in its application to the transitions of supramolecular systems beyond the range of exploration of other computational methods. The computing time in the present method is several orders of magnitude shorter than that required in regular MD or BD simulations. Here, sampling the transition pathway of the intact GroEL (of approximately 8,000 residues) takes less than 255 min CPU time (or about 33 hr real time) on a 2G Hz Linux server. The method has its own limitations, however. The modes predicted by the ANM are those exclusively based on inter-residue contact topology. No specific interactions are taken into consideration. So, the routes predicted here are those selected assuming that mechanical effects purely based on geometry dominate the dynamics. However, specific interactions may become more important as more localized changes are simulated, and these are usually manifested by changes in side chain reorientations, which are beyond the range of *a*ANM calculations. We note in particular that there are well-defined ways to define transition rates through reactive flux theory [78], optimize reaction coordinates and estimate reaction rate coefficients (see for example [79]), which are not addressed in the present study.

Finally, from a broader perspective, it is worth noting that this type of calculation is possible only to the extent that the conformational changes intrinsically favored by the overall architecture comply with those required to achieve the biological function (or the allosteric cycle, here). In principle, the configurations are defined in a *3N*-dimensional space, defined by *3N*-coordinates (provided that we adopt a coarse-grained description of *N* position vectors, one per residue). Here, we let the molecule move in the subspace of 5–6 coordinates only predicted by the ANM, and we can see that more than ½ of the trajectory along the conformational space is traversed by the molecule by simply moving along these soft coordinates. High frequency modes need not be recruited until the energy barrier is approached. The conformational changes are therefore effectuated to a large extent through low frequency modes, which are the least expensive (from energetic point of view), or the most favorable (from entropic point of view) modes among a multitude (3*N-6* of them) of possible modes/directions of reconfiguration. This brings up the issue of a possible evolutionary selection of 3-dimensional structures (or inter-residue contact topologies) whose energy landscape is most conducive to functional changes in conformation. The selection of a conformational changes entropically favored by the structure, in order to achieve biological function, appears to be a common property of proteins, as also evidenced for ubiquitin in a recent study [80].

## Materials and Methods

### Materials

The following GroEL structures deposited in the Protein Data Bank (PDB) [81] have been considered in the present study (Figure 1): (A) 1GR5; the apo-form of GroEL, representative of the T/T state. (B) 1GRU; identified with the R″/R state; it contains seven ADPs and seven ATPs bound to the *cis* and *trans* rings, respectively. All these structures were determined by cryo-electron microscopy by Saibil and coworkers [15],[68],[82]. The structures being compared (the end points and intermediates at each step) were optimally superimposed at their α-carbons using Kabsch method [83] prior to implementing the *a*ANM method.

### *a*ANM method and parameters

To explore the transition pathway between the initial and final conformations of subunit A, a series of intermediate conformations has been generated upon successive deformations of both end structures that were regularly updated. The directions of deformations were determined by implementing the deformations along the directions of dominant ANM modes accessible to the intermediate states. The contribution of the *i^th^* normal mode to the mean-square fluctuations of residues scales with the inverse eigenvalue of the Hessian ***H*** characteristic of the energy landscape near the equilibrium state; and the eigenvalues, in turn, scale with the squared frequency of the normal modes, hence the dominant role of low frequency modes in shaping the equilibrium dynamics [42].

The *a*ANM scheme involves two parameters, *F_min_* and *f*, which, in turn, determine *m_A_* ^(*k*)^, *m_B_* ^(*k*)^, *s_A_* ^(*k*)^ and *s_B_* ^(*k*)^. The dependence of *m_A_* ^(*k*)^ and *m_B_* ^(*k*)^ on *F_min_* has been described in the Theory. As to *s_A_* ^(*k*)^ and *s_B_* ^(*k*)^, these are determined as follows: first we evaluate the values *s_Am_* ^(*k*)^ and *s_Bm_* ^(*k*)^ that minimize |*d* ^(*k*)^|. Note that there is a unique combination of these parameters (or the sizes |
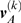
| and |
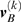
| instantaneous displacements) which leads to the minimal |*d* ^(*k*)^|. However, the step sizes that meet this criterion may be too large to be extrapolated using a quadratic approximation away from the energy minimum, and the energy cost of the induced deformations may be excessively high and not accurately accounted for by the harmonic approximation. Instead, we take a fraction *f* of the calculated values, i.e., we implement displacements that scale with *fs_Am_* ^(*k*)^ and *fs_Bm_* ^(*k*)^. Calculations repeated with different *f* values support the use of relatively small values for two reasons: First, the smaller steps avoid unphysical distortions in the structure (see Supporting Information); second, if a larger displacement along a given mode direction is more productive, the same mode is selected in the succeeding iteration (see for example how modes 1, 3, 4 etc. are selected multiple times at the initial steps of the trajectories in Table 1.) Therefore, in principle, we would like to take steps that are as small as possible. However, the simulations become increasingly time-consuming (due to larger number of iterations) with decreasing step size. Our calculations suggest that the value *f* = 0.2 leads to sufficiently small step sizes along with computationally tractable iterations.

Finally, instead of picking a fixed *F_min_* throughout the entire trajectories, *F_min_* can be chosen dynamically, 
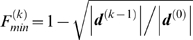
 (for *k*>1). In the first iteration, only the first dominant mode is used to deform the system. The results with this choice of dynamic *F_min_* ^(*k*)^ were found to yield results comparable to *F_min_* = 0.4−0.5 and SDP results (see Figure S6). The use of this empirical relationship has the advantage of eliminating the variable *F_min_* from the *a*ANM algorithm.

### Potential energy function and parameters

We adopt a double minima quadratic energy function [49]

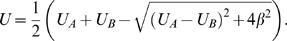
(7)


Here the parameter *β* sets the height (and smoothness) of the energy barrier, and the potentials *U_A_* and *U_B_* are defined in the ANM in terms of a uniform force constant γ as [52]

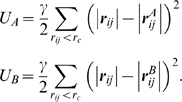
(8)


Here *|* ***r*** *_ij_|* is the instantaneous distance between residues *i* and *j* (based on α-carbons), *|* ***r*** *_ij_^A^|* is the equilibrium distance in conformation A, and the summations are performed over all pairs of residues located within a cutoff distance *r_c_*. The *a*ANM trajectories were generated using Eq. (8) in each iteration by assuming that ***r*** *_ij_^A^* and ***r*** *_ij_^B^* are the final conformations of the previous iteration. Eq. (7) has been used for evaluating the energy of the generated conformations and for performing SDP calculations. We adopted the value *β* = 10 kcal/mol and verified that changes in *β* within one order of magnitude do not affect the results. Nevertheless, the energies reported below provide information on the shape, rather than absolute values, of the energy profile. The force constant is taken as 0.7 kcal/(mol·Å^2^), and the cut-off distance as *r_c_* = 13.0 Å consistent with previous assessment of optimal ANM parameters [55].

### Steepest descent path (SDP)

The SDP between the conformations 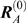 and 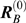 is calculated by numerical minimization of the integral
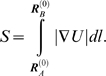
(9)


The product 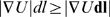, therefore 
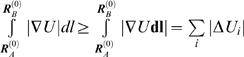
, where 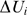 is potential energy difference in the *i* ^th^ monotonic part of the trajectory. The sum 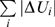 is minimal if the molecule undergoes a transition from 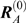 to 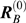 through the lowest energy barrier; moreover, the terms in the last inequality are equal if and only if the trajectory proceeds parallel to the potential gradient. Therefore SDP is the unique minimum of the boundary value integral in Eq. (9). To find a path that minimizes *S* (i.e., the SDP), we search for the global minimum of a discrete approximation
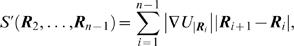
(10)subject to the constraint that successive 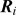
's are equally spaced along the trajectory. The critical advantage of this algorithm is that it remains stable and calculates qualitatively reasonable trajectories even when the distances between 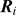
's are large. The minimization of 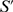 is accomplished by simulated annealing. For further details see [29] and [63].

## Supporting Information

Figure S1 Contribution of low frequency modes to the conformational changes undergone by a single subunit during the cycle T→R→R″→T.
(0.84 MB DOC)

Figure S2 Contribution of low frequency modes to the conformational changes undergone by the intact chaperonin during the cycle T/T→R/T, R/T→R′/T and R″/R→T/R.
(0.79 MB DOC)

Figure S3 Distortion in backbone bonds during conformational transitions.
(0.32 MB DOC)

Figure S4 Time evolution of salt bridges.
(0.78 MB DOC)

Figure S5 Robustness of the broken/formed native contacts near the transition point.
(0.41 MB DOC)

Figure S6 Comparison with the paths predicted by MinActionPath, SDP and Interpolation.
(0.10 MB DOC)

Table S1 Distortion in backbone bonds during conformational transitions.
(0.03 MB DOC)

Text S1 Relationship between cumulative correlation cosine and the angle formed by ***d*** ^(*k*)^ and combined eigenvectors (derivation of Eq.(6)).
(0.10 MB DOC)
