# Supplementary material for: Allosteric Transitions of Supramolecular Systems Explored by Network Models: Application to Chaperonin GroEL
Source: PLoS Comput Biol. 2009 Apr 17;5(4):e1000360. doi: 10.1371/journal.pcbi.1000360 (PMC2664929; doi:10.1371/journal.pcbi.1000360)
Supplement: Figure S1 — Contribution of low frequency modes to the conformational changes undergone by a single subunit during the cycle T→R→R″→T. (0.84 MB DOC) [file pcbi.1000360.s001.doc]

**Supplementary Material**

2(a). Contribution of low frequency modes to the conformational changes undergone by a single subunit during the cycle T  R  R”  T

**Figure S1** displays the decrease in the $RMSD(R_{A}^{(k)},R_{B}^{(k)})$= |***d***(*^k^*)|/N between the end states of each of the three successive steps, T 🡪 R, R 🡪 R” and R”🡪 T, of the allosteric cycle, as the GroEL subunit undergoes a conformational change along the slowest modes. The panels on the right refer to the forward transitions, and those on the right are the corresponding reverse transitions. Each panel contains three curves, corresponding to the reconfiguration along the slowest ANM mode coordinate (black), along a combination of two slow modes (red) and three slow modes (blue), as indicated by the labels. The RMSDs attained at the end of these trajectories represent the closest distance of approach that can be reached in a single iteration (*k* = 1) of the ANM algorithm upon recruiting the modes indicated in each panel. The final RMSD values are listed in **Table III**. Note that the subunit is subject to ~ 1,500 modes of motion. The predominant role of the slowest 4-5 modes, and in particular the mode 1, is remarkable.

**Figure S1**
