# Supplementary material for: Allosteric Transitions of Supramolecular Systems Explored by Network Models: Application to Chaperonin GroEL
Source: PLoS Comput Biol. 2009 Apr 17;5(4):e1000360. doi: 10.1371/journal.pcbi.1000360 (PMC2664929; doi:10.1371/journal.pcbi.1000360)
Supplement: Figure S2 — Contribution of low frequency modes to the conformational changes undergone by the intact chaperonin during the cycle T/T→R/T, R/T→R′/T and R″/R→T/R. (0.79 MB DOC) [file pcbi.1000360.s002.doc]

**Supplementary Material**

2(b). Contribution of low frequency modes to the conformational changes undergone by the intact chaperonin during the cycle T/T  R/T, R/T  R’/T and R”/R T/R

**Figure S2** is the counterpart of **Figure S1** generated for the intact chaperonin. In this case, we have a total of *3N – 6* = 24,039 modes, given that *N* = 8015 for the GroEL-GroES complex. Note that in the absence of the cap, the number of modes reduces to 21,582 (i.e*. N* = 7196). Many modes in the low frequency regime are degenerate, due to the structural symmetry. Of interest are the non-degenerate slowest modes, which uniformly affect all subunits. The curves displayed in **Figure S2** (and the data reported in **Table IV**) describe the effect of one (black), three (red) and six (blue) non-degenerate modes at the lowest frequency end of the spectrum. Results are shown for the reconfigurations starting from either end of the transition (*left* and *right* panels). In line with the results shown for the single subunit, a handful of non-degenerate modes set is sufficient to generate on-pathway intermediates as evidenced by the RMSD values reached in each case.

**Figure S2**
