# Supplementary material for: Allosteric Transitions of Supramolecular Systems Explored by Network Models: Application to Chaperonin GroEL
Source: PLoS Comput Biol. 2009 Apr 17;5(4):e1000360. doi: 10.1371/journal.pcbi.1000360 (PMC2664929; doi:10.1371/journal.pcbi.1000360)
Supplement: Figure S3 — Distortion in backbone bonds during conformational transitions. (0.32 MB DOC) [file pcbi.1000360.s003.doc]

**Supplementary Material**

3(a). Distortion in backbone bonds during conformational transitions

**Figure S3.** To monitor the possible distortion in backbone bonds along the transition pathways, the C^α^-C^α^ bond lengths at intermediate states were calculated (for single subunit). The error bars indicate the standard deviations from average value. The average bond lengths and their standard deviations for various *F_min_* values with *f* = 0.2 are summarized in **Table S.I**. The *F_min_* in 0.5, 0.6 or 0.7 give almost the same average bond length, 3.81 ± 0.12 Å, which confirmed that the reconfigurations did not induce any unrealistic distortions in backbone bond lengths.

**Figure S3**
