# Supplementary material for: Allosteric Transitions of Supramolecular Systems Explored by Network Models: Application to Chaperonin GroEL
Source: PLoS Comput Biol. 2009 Apr 17;5(4):e1000360. doi: 10.1371/journal.pcbi.1000360 (PMC2664929; doi:10.1371/journal.pcbi.1000360)
Supplement: Figure S4 — Time evolution of salt bridges. (0.78 MB DOC) [file pcbi.1000360.s004.doc]

**Supplementary Material**

4. Time evolution of salt bridges

The curves below are the counterparts of those presented in Figure 8, replotted as a function of time. The time dependence is deduced by normalizing one of the curves in each panel, labeled ‘reference curve’, with respect to those presented in the Figures 4 and 5 of ref. [18] (Hyeon et al, 2006).

|  |  |
| --- | --- |

**Figure S4**
