# Supplementary material for: Allosteric Transitions of Supramolecular Systems Explored by Network Models: Application to Chaperonin GroEL
Source: PLoS Comput Biol. 2009 Apr 17;5(4):e1000360. doi: 10.1371/journal.pcbi.1000360 (PMC2664929; doi:10.1371/journal.pcbi.1000360)
Supplement: Figure S5 — Robustness of the broken/formed native contacts near the transition point. (0.41 MB DOC) [file pcbi.1000360.s005.doc]

**Supplementary Material**

5. **Robustness of the broken/formed native contacts near the transition point**

The figure below shows the evolution of native contacts along the reaction coordinate as a GroEL subunit undergoes a conformational change from R” to T state, along various pathways corresponding to different *F_min_* values. The number of native contacts that are broken (left panels) and formed (right panels) are plotted therein as a function of the reaction coordinate. Each bar represents the change of the number of native contacts at a given step of the *a*ANM method.

**Figure S5**
