# Supplementary material for: Allosteric Transitions of Supramolecular Systems Explored by Network Models: Application to Chaperonin GroEL
Source: PLoS Comput Biol. 2009 Apr 17;5(4):e1000360. doi: 10.1371/journal.pcbi.1000360 (PMC2664929; doi:10.1371/journal.pcbi.1000360)
Supplement: Figure S6 — Comparison with the paths predicted by MinActionPath, SDP and Interpolation. (0.10 MB DOC) [file pcbi.1000360.s006.doc]

**Supplementary Material**

**6. Comparison with the paths predicted by MinActionPath, SDP and Interpolation**

The transition pathway between 1GRU_A(**R**”) and 1GR5_A(**T**) was also generated with MinActionPath(MAP) web based server (ref. [31] Franklin et al, 2007). With the same energy function (Eq.7), the energies from various methods are compared below in the top panel of **Figure S6**. The bottom panel displays the departure from direct interpolation for all generated trajectories. The curve labeled *Dyn* is evaluated by adopting the iteration dependent $F_{min}^{(k)}=1-\sqrt{d^{(k-1)}/d^{(0)}}$ expression given in the Methods. The legends in numbers refer to *a*ANM with respective *F_min_* values.

**Figure S6**
