# Supplementary material for: Allosteric Transitions of Supramolecular Systems Explored by Network Models: Application to Chaperonin GroEL
Source: PLoS Comput Biol. 2009 Apr 17;5(4):e1000360. doi: 10.1371/journal.pcbi.1000360 (PMC2664929; doi:10.1371/journal.pcbi.1000360)
Supplement: Table S1 — Distortion in backbone bonds during conformational transitions. (0.03 MB DOC) [file pcbi.1000360.s007.doc]

**Supplementary Material**

3(b). Distortion in backbone bonds during conformational transitions

To monitor the possible distortion in backbone bonds along the transition pathways, the C^α^-C^α^ bond lengths at intermediate states were calculated (for single subunit). The results are summarized in **Table S.I** (2^ed^ column) and **Figure S3**. We also monitored the non-bonded interactions (3^rd^ column in **Table S.I**) during the structural changes to confirm that no steric clashes (non-bonded C^α^-C^α^ distances shorter than 3.4Å) occurred at any stage, including the close neighborhood of the transition state where notable redistributions in non-bonded contacts took place (see main text).

**Table S.I Backbone bond lengths and shortest non-bonded distances in *a*ANM**

| ***F_min_*** | **Average backbone bond lengths and their standard deviation (Å)** | **Shortest non-bonded distances (Å)** |
| --- | --- | --- |
| 0.4 | 3.82 ± 0.14 | 3.50 |
| 0.5 | 3.82 ± 0.12 | 3.43 |
| 0.6 | 3.81 ± 0.12 | 3.54 |
| 0.7 | 3.81 ± 0.12 | 3.55 |
