# Supplementary material for: Allosteric Transitions of Supramolecular Systems Explored by Network Models: Application to Chaperonin GroEL
Source: PLoS Comput Biol. 2009 Apr 17;5(4):e1000360. doi: 10.1371/journal.pcbi.1000360 (PMC2664929; doi:10.1371/journal.pcbi.1000360)
Supplement: Text S1 — Relationship between cumulative correlation cosine and the angle formed by d (k) and combined eigenvectors (derivation of Eq.(6)). (0.10 MB DOC) [file pcbi.1000360.s008.doc]

**Supplementary Material**

# **1. Relationship between cumulative correlation cosine and the angle formed by d^(k)^ and combined eigenvectors (derivation of Eq.**(6)**)**

The instantaneous distance vector $d^{(k)}$ can be expressed as:

$$d^{(k)}=R_{B}^{(k)}-R_{A}^{(k)}=\sum_{i=1}^{3N-6} \alpha_{i}u_{iA}^{(k)}$$

The instantaneous deformation vector $v_{A}^{(k)}$ can, in turn, be written as the summation of weighted eigenvectors (see Eq.(4)) as

$$v_{A}^{(k)}=R_{A}^{(k)}-R_{A}^{(k-1)}=s_{A}^{(k)}\sum_{j=1}^{m_{A}^{(k)}} \alpha_{jA}^{(k)} u_{jA}^{(k)}$$

We will conveniently omit the subscript *A* and superscript *k* in the following, as the derivation holds for all steps (*k*) and starting conformation (A or B). The dot product of the vectors $d$and $v$can be written in terms of the summations over nonzero modes as

$$d\cdot v=\left(\sum_{i=1}^{3N-6} \alpha_{i}u_{i}\right)\cdot\left(s\sum_{j=1}^{m} \alpha_{j}u_{j}\right)=s\sum_{j=1}^{m} \alpha_{i}^{2}\left|u_{i}\right|^{2}$$

The equality follows from the orthogonality of the eigenvectors, i.e., $u_{i}\cdot u_{j}=\delta_{ij}$. Also, since the eigenvectors are normalized,$\left|u_{i}\right|=1$, we obtain

$$d\cdot v=s\sum_{i=1}^{m} \alpha_{i}^{2}$$

Using the definitions$\alpha_{i}=\left|d\right|cos(d,u_{i})$ and $F(m)=\sum_{i=1}^{m} \left(cos(d,u_{i})\right)^{2}$in Eq., we obtain

$$d\cdot v=s\sum_{i=1}^{m} \left(\left|d\right|cos(d,u_{i})\right)^{2}=s\left|d\right|^{2}F(m)$$

On the other hand,

$$\left|v\right|^{2}=\left(s\sum_{i=1}^{m} \alpha_{i}u_{i}\right)\cdot\left(s\sum_{i=1}^{m} \alpha_{i}u_{i}\right)=s^{2}\sum_{i=1}^{m} \alpha_{i}^{2}\left|u_{i}\right|^{2}=s^{2}\sum_{i=1}^{m} \alpha_{i}^{2}$$

Here we have utilized both orthogonality and unit magnitude properties of the eigenvectors, $u$. Using the definitions of α*_i_* and *F*(*m*), this equality becomes

$$\left|v\right|^{2}=s^{2}\sum_{i=1}^{m} \left(\left|d\right|cos(d,u_{i})\right)^{2}=s^{2}\left|d\right|^{2}F(m)$$

which implies $\left|v\right|=s\left|d\right|\left[F(m)\right]^{1/2}$. Combining this result with Eq. leads to Eq.(6).
